# Development of EST-SSR markers in flowering Chinese cabbage (*Brassica campestris* L. ssp. *chinensis* var. *utilis* Tsen et Lee) based on *de novo* transcriptomic assemblies

**DOI:** 10.1371/journal.pone.0184736

**Published:** 2017-09-13

**Authors:** Jingfang Chen, Ronghua Li, Yanshi Xia, Guihua Bai, Peiguo Guo, Zhiliang Wang, Hua Zhang, Kadambot H. M. Siddique

**Affiliations:** 1 International Crop Research Center for Stress Resistance, College of Life Sciences, Guangzhou University, Guangzhou, China; 2 Hard Winter Wheat Genetics Research Unit, United States Department of Agriculture–Agricultural Research Service, Manhattan, Kansas, United States of America; 3 Guangzhou Academy of Agricultural Sciences, Guangzhou, China; 4 The UWA Institute of Agriculture and School of Agriculture & Environment, The University of Western Australia, Perth WA, Australia; Youngstown State University, UNITED STATES

## Abstract

Flowering Chinese cabbage is one of the most important vegetable crops in southern China. Genetic improvement of various agronomic traits in this crop is underway to meet high market demand in the region, but the progress is hampered by limited number of molecular markers available in this crop. This study aimed to develop EST-SSR markers from transcriptome sequences generated by next-generation sequencing. RNA-seq of eight cabbage samples identified 48,975 unigenes. Of these unigenes, 23,267 were annotated in 56 gene ontology (GO) categories, 6,033 were mapped to 131 KEGG pathways, and 7,825 were assigned to clusters of orthologous groups (COGs). From the unigenes, 8,165 EST-SSR loci were identified and 98.57% of them were 1–3 nucleotide repeats with 14.32%, 41.08% and 43.17% of mono-, di- and tri-nucleotide repeats, respectively. Fifty-eight types of motifs were identified with A/T, AG/CT, AT/AT, AC/GT, AAG/CTT and AGG/CCT the most abundant. The lengths of repeated nucleotide sequences in all SSR loci ranged from 12 to 60 bp, with most (88.51%) under 20 bp. Among 170 primer pairs were randomly selected from a total of 4,912 SSR primers we designed, 48 yielded unambiguously polymorphic bands with high reproducibility. Cluster analysis using 48 SSRs classified 34 flowering Chinese cabbage cultivars into three groups. A large number of EST-SSR markers identified in this study will facilitate marker-assisted selection in the breeding programs of flowering Chinese cabbage.

## Introduction

Molecular markers are widely used in plant science for various applications. Due to quick advancements in molecular biology, the marker technologies rapidly evolved from restriction fragment length polymorphisms (RFLP) to simple sequence repeats (SSR) and single nucleotide polymorphisms (SNP). SSR markers are tandem repeats of 1–6 bp of nucleotides that are widespread in whole genomes of different species. SSR can be divided into genomic SSR and expressed-sequence-tag-based SSR (EST-SSR). SSR markers have many advantages over other markers including a high level of polymorphism, co-dominant inheritance, and high specificity and repeatability [1, 2]. They are widely used to analyze genetic diversity and biological evolution, construct genetic linkage maps, and map quantitative trait loci (QTLs) and for marker-assisted breeding [3]. SSR markers have been developed in many crop species and applied to genetic improvement of these species, including rice bean, maize, and sesame [4–6].

Flowering Chinese cabbage (*Brassica campestris* L. ssp. *chinensis* var. *utilis* Tsen et Lee) is an important vegetable crop in southern China. It is a popular vegetable crop for its rich nutritional value and favorable taste. Although flowering Chinese cabbage research has recently focused more on agronomic traits, physiological properties and genetic diversity [7], transcriptome analysis and development of molecular markers for breeding are still in its infancy [8]. Exploring new SSR markers in flowering Chinese cabbage will facilitate genetic and molecular studies of this species. The conventional method for SSR development involves constructing a genome or EST library, sequencing all clones in the library, and identifying SSRs. SSR primers are then designed according to the bilateral sequences of the identified SSR loci [2]. However, this method is labor intensive and produces relatively a few SSRs [6]. Recent developments in genome sequencing and *de novo* assembly provide opportunities to simultaneously develop a large number of SSR markers. RNA-sequencing (RNA-seq), using next generation sequencing technology, makes it possible to develop a large number of SSR markers in transcribed regions of a genome. Using RNA-seq to develop SSRs has advantages of relatively cost effective and direct use of expressed gene sequences [6]. Development of SSR markers with genetic information using RNA-seq and their application in diversity analysis have been reported in many species including Chinese cabbage, radish, carnation and moth orchid [9–12], but not in flowering Chinese cabbage. Although both flowering Chinese cabbage and Chinese cabbage belong to the *Brassica rapa* genome, their morphological traits and planting areas differ significantly. Breeders and researchers of flowering Chinese cabbage consider Chinese cabbage as an alien species in breeding program. The available SSR markers from *Brassica rapa* including Chinese cabbage can be transferred to flowering Chinese cabbage, but the efficiency of transferability is less than 37.6% [8]. In addition, potential SSRs identified in Chinese cabbage by Ding et al. [9] were not further detected and utilized. Therefore, developing SSR markers using RNA-seq data is needed in flowering Chinese cabbage.

As an important abiotic stress for flowering Chinese cabbage, heat stress has serious impacts on its quality and production. Based on experiments investigating genes responsive to heat stress at the seedling stage in flowering Chinese cabbage by using RNA-seq, transcriptome data of four genotypes under control and heat stress conditions were collected, and 8,165 SSR loci were identified. We designed primers for 4,912 SSRs, randomly selected 170 SSRs for validation in four flowering Chinese cabbage genotypes, and analyzed 48 polymorphic SSRs in a diversity panel of 34 cultivars.

## Material and methods

### Plant materials

Four flowering Chinese cabbage genotypes, Sijiu-19, Youlv 501, 3T-6 and Liuye 50, provided by Guangzhou Academy of Agricultural Sciences, Guangzhou, China, were sown in pots and grown in a growth chamber with temperatures set at 28/22°C for 14/10 h (day/night). Each genotype had six pots with 3 seedlings per pot. At five-leaf stage, three pots per genotype were moved to a different growth chamber for 6 h heat stress treatment at 38°C/29°C (14/10 h). After heat stress, the first fully expanded leaves of selected plants were harvested, together with the plants at the same stage in the control groups, snap frozen in liquid nitrogen, and stored at –80°C for RNA extraction. Young leaves of all the four genotypes were also harvested for DNA isolation.

### DNA and RNA isolation

Genomic DNA was extracted using a modified CTAB method [13] and total RNA was isolated using a Trizol RNA extraction kit (Invitrogen, USA) [14]. The concentration and quality of extracted RNA and DNA were evaluated using a microplate spectrophotometer (BioTek Company, USA) and 1% agarose gel electrophoresis, respectively. DNA was diluted to 10 ng·μl^–1^ for SSR analysis.

### Sequence analysis

The total RNA with equal quantity was pooled from each sample, the mRNA was purified from the pooled RNA using Oligo dT attached magnetic beads, fragmented, and used to synthesize the cDNA. Double-stranded cDNA was purified and end-repaired, then single nucleotide adenine and the sequence adaptors were added to the cDNA fragments for ligation. The target fragments were PCR amplified for construction of cDNA libraries. The cDNA libraries were sequenced using an Illumina HiSeq2000 platform (Annoroad Gene Company, Beijing, China).

Raw data was filtered by removing the adaptors, low quality reads (Phred quality score ≤ 19) and reads with unknown bases. De novo assembly of full-length transcripts was performed using Trinity as decribed by Grabherr et al. [15] with three steps: 1) contigs were assembled by Inchworm software; 2) Bruijn graphs were built using Chrysalis software; 3) alternatively spliced transcripts were obtained and paralogous transcripts were separated through Butterfly software.

### Functional annotation of unigenes

To annotate unigenes, putative protein sequences were predicted from all unigenes using TransDecoder tools (http://transdecoder.sourceforge.net/). The functions of putative protein sequences were annotated using Trinotate tools (http://trinotate.github.io/) against NCBI non-redundant (Nr) (http://www.ncbi.nlm.nih.gov/genbank/), Swiss-Prot (http://www.uniprot.org/), and Kyoto Encyclopedia of Genes and Genomes (KEGG) pathway (http://www.genome.jp/kegg/pathway.html) databases, using the E value ≤ 1e^-5^. GO (Gene Ontology) functional classifications (http://www.geneontology.org/) including molecular function, biological process, and cellular component clusters were obtained by using Blast2GO [16]. Furthermore, all unigenes were aligned to the COG (clusters of orthologous groups) (http://www.ncbi.nlm.nih.gov/COG) to predict their possible functions.

### SSR primer design and SSR marker screening

SSRs from unigenes were identified using the MIcroSAtellite identification tool (MISA, http://pgrc.ipk-gatersleben.de/misa/) [17]. Repeat sequence motifs included one to six nucleotides with repetitions of 12, 6, 5, 5, 5 and 5.

SSR primers were designed based on the bilateral sequence of SSR using BatchPrimer3 v1.0 software at http://probes.pw.usda.gov/cgi-bin/batchprimer3/batchprimer3.cgi. Lengths of the primers ranged from 18–25 bp with most around 20 bp, and melting temperatures (Tm) ranged from 55–60°C. PCR products ranged from 100–300 bp. The percentages of GC content in the designed primers ranged from 25–65% with most around 50%. Other parameters were set as default. All primers were synthesized by the Sangon Biotech Company (Shanghai, China).

### Validation and application of SSR markers

Four genotypes were used to validate designed SSR primers. The PCR reactions were carried out in a 10μl volume containing 20 ng of template DNA, 1×PCR buffer, 2.0 mM of MgCl_2_, 0.25 μM of each forward and reverse primer, 0.25 mM of dNTPs, 0.75 u of *Taq* DNA polymerase. PCR amplification was performed using an ABI9700 PCR cycler (Applied Biosystems, USA) using the following program: 94°C pre-denaturation for 5 min, 35 cycles of 94°C denaturation for 45 s, varied annealing temperatures ranging from 55–60°C with different primers for 45 s, 72°C extension for 1 min, and a final step at 72°C for 10 min. The PCR products were visualized in a 6% polyacrylamide gel after silver staining [18].

Thirty-four flowering Chinese cabbage cultivars (S1 Table) were genotyped using 48 validated SSRs. Polymorphic information content (PIC) was calculated using PIC_Calc 0.6 software [19], and observed heterozygosities (*Ho*) and expected heterozygosities (*He*) were obtained by using the POPGENE 1.32 software [20]. Genetic similarity of these accessions was analyzed using NTsys 2.10e software [21]. Based on the data of genetic similarity coefficients, a dendrogram tree was constructed using the unweighted pair-groups from the UPGMA module. The reliability of dendrogram was tested through bootstrap analysis based on 1000 trials using Phylip v3.69 [22].

## Results

### Sequencing data and *de novo* assembly

**Fig 1:**
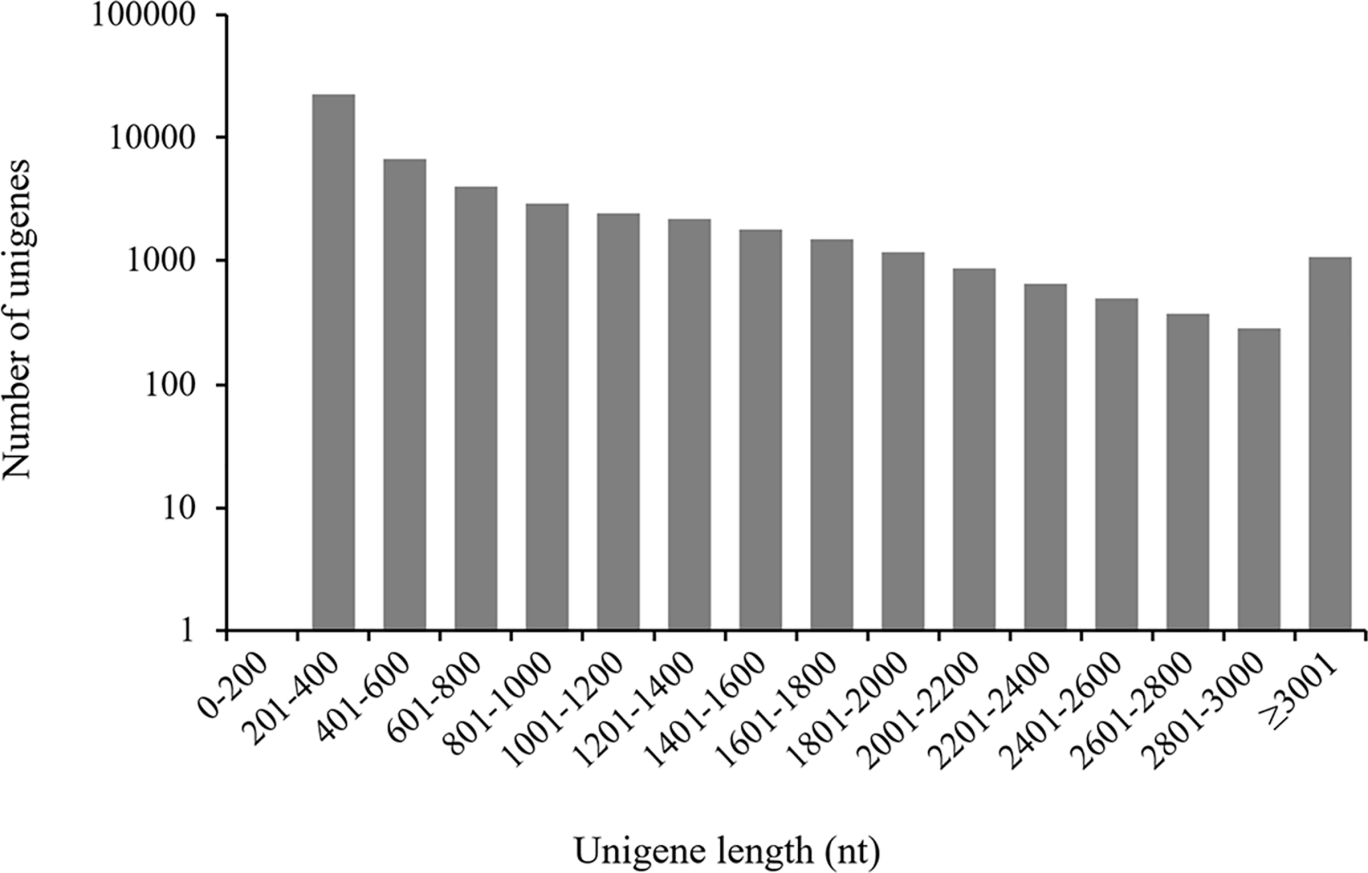
Length distribution of assembled unigenes from the transcriptome of flowering Chinese cabbage.

A total of 2.95 × 10^8^ clean reads with 36.9 Gb sequence were generated from eight samples. The sequence data were deposited into the NCBI SRA database under the accession number SRP114731 (https://www.ncbi.nlm.nih.gov/Traces/study/?acc=SRP114731). A total of 48,975 unigenes with 38.2 Mb sequence were obtained with an average length of 779 bp per unigene. N50 and N90 were 1,317 and 301 bp, respectively, which represent the sequence length of the smallest contig in the set that contains the fewest (largest) contig whose combined length produces at least 50% or 90% of the assembly, respectively [23]. Near half of the unigenes (22,677 or 46.30%) are 201–400 bp, 6,699 unigenes (13.68%) are 401 to 600 bp, and 19,599 unigenes (40.02%) are over 600 bp with 12,733 of them over 1,000 bp (Fig 1). The number of unigenes increased with the decrease in the sequence lengths of unigenes. This Transcriptome Shotgun Assembly project has been deposited at DDBJ/EMBL/GenBank under the accession GFUS00000000. The version described in this paper is the first version, GFUS01000000.

### Unigene annotation

Of the 48,975 unigenes, 37,494 (76.56%) and 24,524 (50.07%) showed significant similarity to proteins in the Nr and Swiss-Prot databases, respectively, and 24,406 (49.83%) were found in both databases. However, 6,864 (14.02%) couldn’t be located in any existing databases (S2 Table).

Ground on Nr annotation, 23,267 (47.51%) unigenes showed functions in biological process, cellular component and molecular function, which can be further classified into 56 functional groups in Gene Ontology (GO) (S1 Fig). The seven major GO groups were cell (14,977, 64.37%), cell part (14,977, 64.37%), binding (11,222, 48.23%), cellular process (10,747, 46.19%), organelle (9,952, 42.77%), single-organism process (8,455, 36.34%) and metabolic process (7,762, 33.36%). The smallest groups, with only one unigene assigned, included biological phase, detoxification, virion part and synapse part.

**Fig 2:**
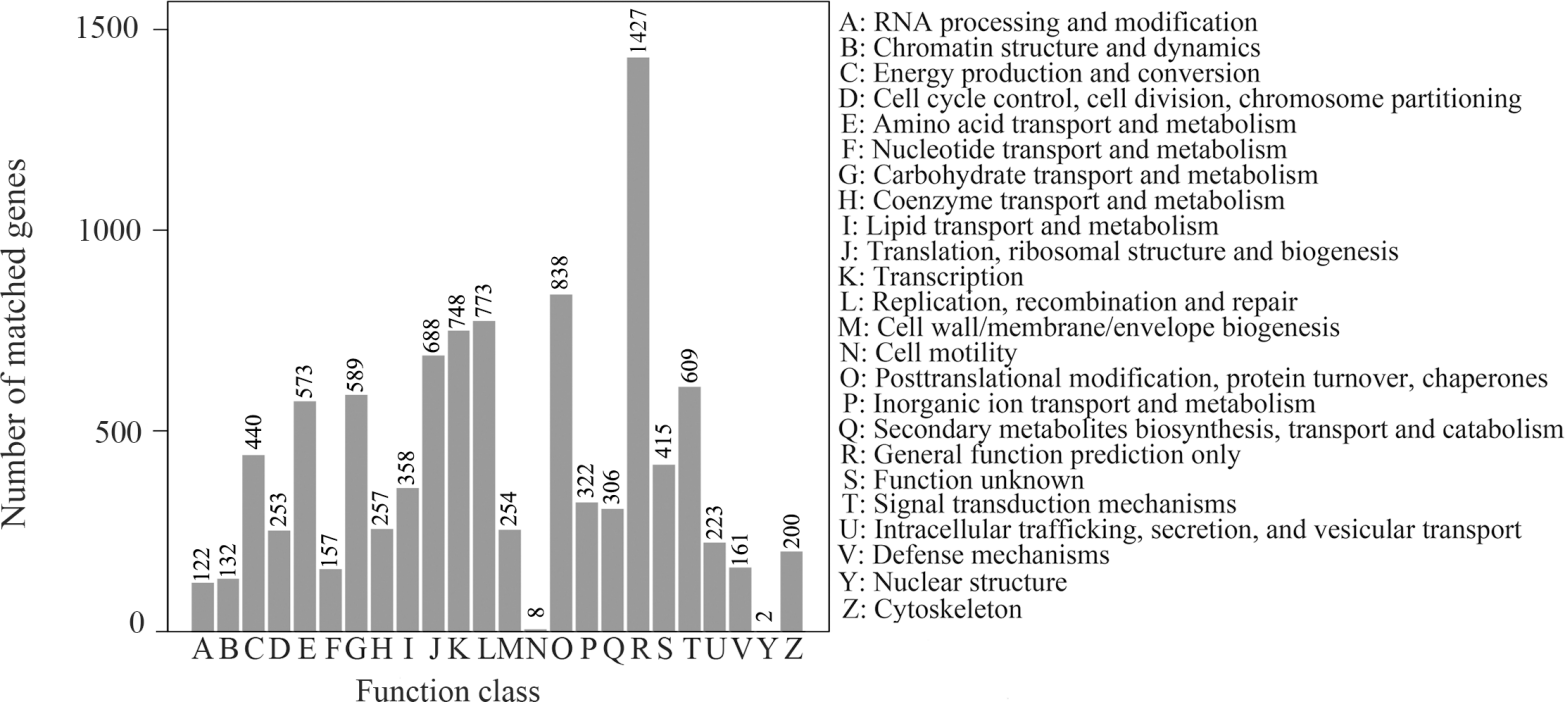
Classification of the assembled unigenes by clusters of orthologous groups (COG) analysis.

In addition, functional prediction and classification of all unigenes were performed against the COG database (Fig 2). Of the 48,975 unigenes, 7,825 (15.98%) were classified into 24 molecular functional families. Among them the five predominant families were ‘general function prediction only’ (1,427, 18.24%), ‘post-translational modification, protein turnover, chaperones’ (838, 10.71%), ‘replication, recombination and repair’ (773, 9.88%), ‘transcription’ (748, 9.56%) and ‘translation, and ribosomal structure and biogenesis’ (688, 8.79%). The smallest families, with only eight and two unigenes assigned, were ‘cell motility’ and ‘nuclear structure’, respectively. The proportions of other families ranged from 1.6% to 7.8%.

The identified 48,975 unigenes were further analyzed in the KEGG database, and 6,033 (12.32%) of them had significant matches in 5 categories including metabolism (3,013, 49.94%), genetic information processing (2,141, 35.49%), environmental information processing (538, 8.92%), cellular processes (499, 8.27%) and organismal systems (359, 5.95%). These categories related to 131 KEGG pathways with 1 to 402 unigenes that were involved in an individual pathway (S3 Table). Of these KEGG pathways, plant hormone signal transduction (402, 6.66%) had the highest unigene hits, followed by biosynthesis of amino acids (367, 6.08%), carbon metabolism (357, 5.92%) and ribosome (306, 5.07%). All other pathways had fewer than 300 unigenes involved (Table 1).

**Table 1:** The 20 KEGG pathways that have the highest unigene numbers.

Number	Name of pathway	No. of unigenes	Pathway ID
1	Plant hormone signal transduction	402 (6.66%)	ko04075
2	Biosynthesis of amino acids	367 (6.08%)	ko01230
3	Carbon metabolism	357 (5.92%)	ko01200
4	Ribosome	306(5.07%)	ko03010
5	Plant-pathogen interaction	296 (4.91%)	ko04626
6	Protein processing in endoplasmic reticulum	278 (4.61%)	ko04141
7	Spliceosome	251 (4.16%)	ko03040
8	Starch and sucrose metabolism	247 (4.09%)	ko00500
9	Ubiquitin mediated proteolysis	240 (3.98%)	ko04120
10	Purine metabolism	209 (3.46%)	ko00230
11	Endocytosis	206 (3.41%)	ko04144
12	RNA degradation	186 (3.08%)	ko03018
13	Amino sugar and nucleotide sugar metabolism	184 (3.05%)	ko00520
14	RNA transport	179 (2.97%)	ko03013
15	mRNA surveillance pathway	167 (2.77%)	ko03015
16	Oxidative phosphorylation	167 (2.77%)	ko00190
17	Pyrimidine metabolism	167 (2.77%)	ko00240
18	Phenylpropanoid biosynthesis	163 (2.70%)	ko00940
19	Cysteine and methionine metabolism	158 (2.62%)	ko00270
20	Peroxisome	151 (2.50%)	ko04146

### Frequency and distribution of SSR loci in flowering Chinese cabbage transcriptome

Screening the 48,975 unigenes, 8,165 SSR loci (distribution frequency of 16.68%) were discovered from 6,778 unigenes (S4 Table). Among these unigenes, 1,149 contained more than 1 SSR locus, and 413 SSRs presented in compound formation. In an average, every 4.67 kb sequences have an SSR locus in the flowering Chinese cabbage transcriptome.

Among the six types of SSR loci identified, trinucleotide repeats were the most frequent (7.2%), followed by dinucleotide (6.85%), whereas tetra, penta and hexanucleotide repeats were the least frequent (Table 2). The mean distances of the mono to hexa SSR loci types across the 38.17 Mb of unigene sequences were negatively correlated with their distribution frequency. Hexa and mononucleotide repeats had the longest (3,816.93 kb) and the shortest (10.83 kb) mean distances, respectively. The discovered SSR repeat sequence length per locus was 15.47 bp, with hexa SSRs being the longest (37.2 bp) and mononucleotide the shortest (13.93bp).

**Table 2:** Distribution of SSR loci among the transcriptome of flowering Chinese cabbage.

SSR type	Number	Distribution frequency (%)	Average distance (kb)	Mean length (bp)	Percentage (%)	Number of motifs
Mono-	1,169	2.39	32.65	13.93	14.32	2
Di-	3,354	6.85	11.38	14.72	41.08	4
Tri-	3,525	7.2	10.83	16.43	43.17	10
Tetra-	92	0.19	414.88	21.13	1.13	20
Penta-	15	0.03	2,544.62	26.33	0.18	12
Hexa-	10	0.02	3,816.93	37.2	0.12	10
Total	8,165	16.68	6,831.29	129.74	100	58

**Fig 3:**
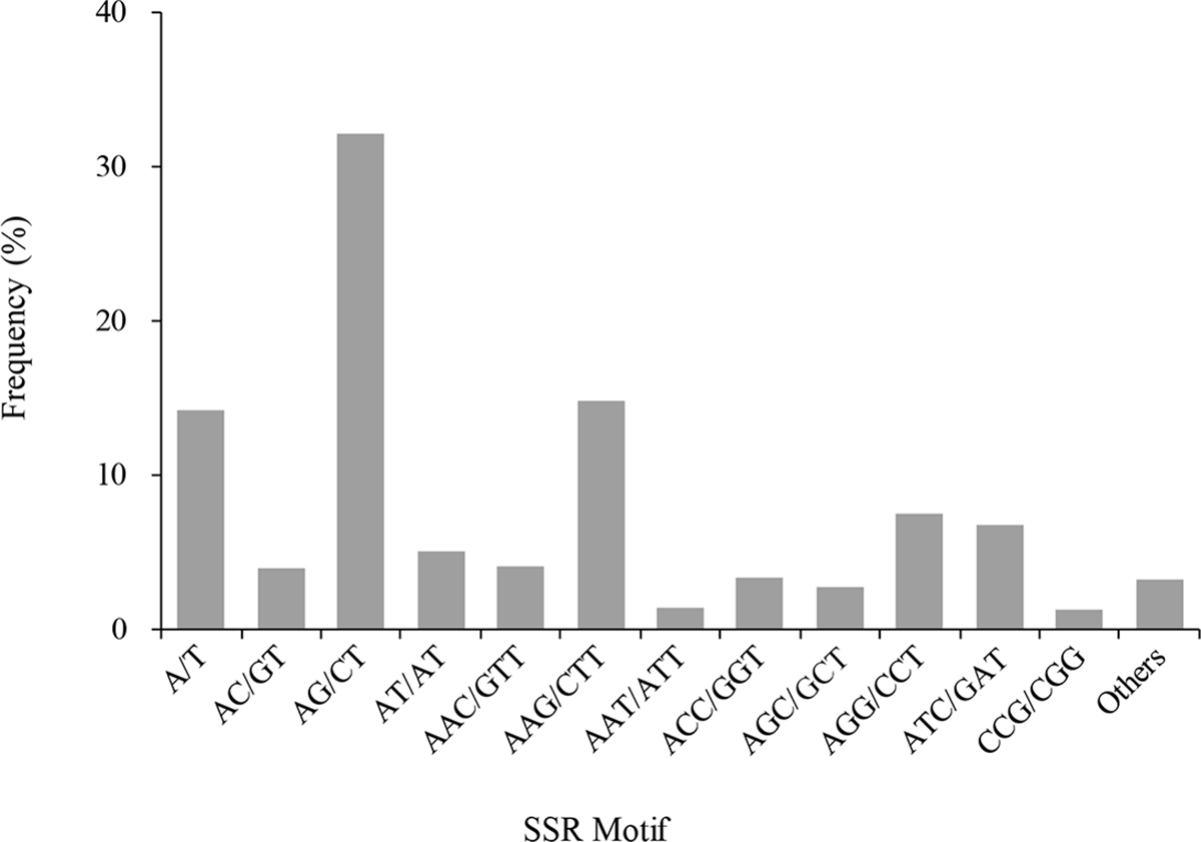
Distribution of SSR motifs in the flowering Chinese cabbage transcriptome.

Among the 8,165 SSR loci identified, the percentage of trinucleotide repeats was the highest (43.17%), followed by dinucleotide repeats (41.08%), mononucleotide repeats (14.32%), tetranucleotide repeats (1.13%), pentanucleotide repeats (0.18%) and hexanucleotide repeats (0.12%) (Table 2). The six SSR types consisted of 58 motifs of repeat sequences (S5 Table) including 20 tetranucleotide, 12 pentanucleotide, 10 trinucleotide, 10 hexanucleotide, 4 dinucleotide and two mononucleotide repeats. The most frequent motif type in the SSR loci was AG/CT (32.09%), followed by AAG/CTT (14.78%) and A/T (14.12%) (Fig 3). The remaining motifs were all less than 10%.

### SSR validation

**Fig 4:**
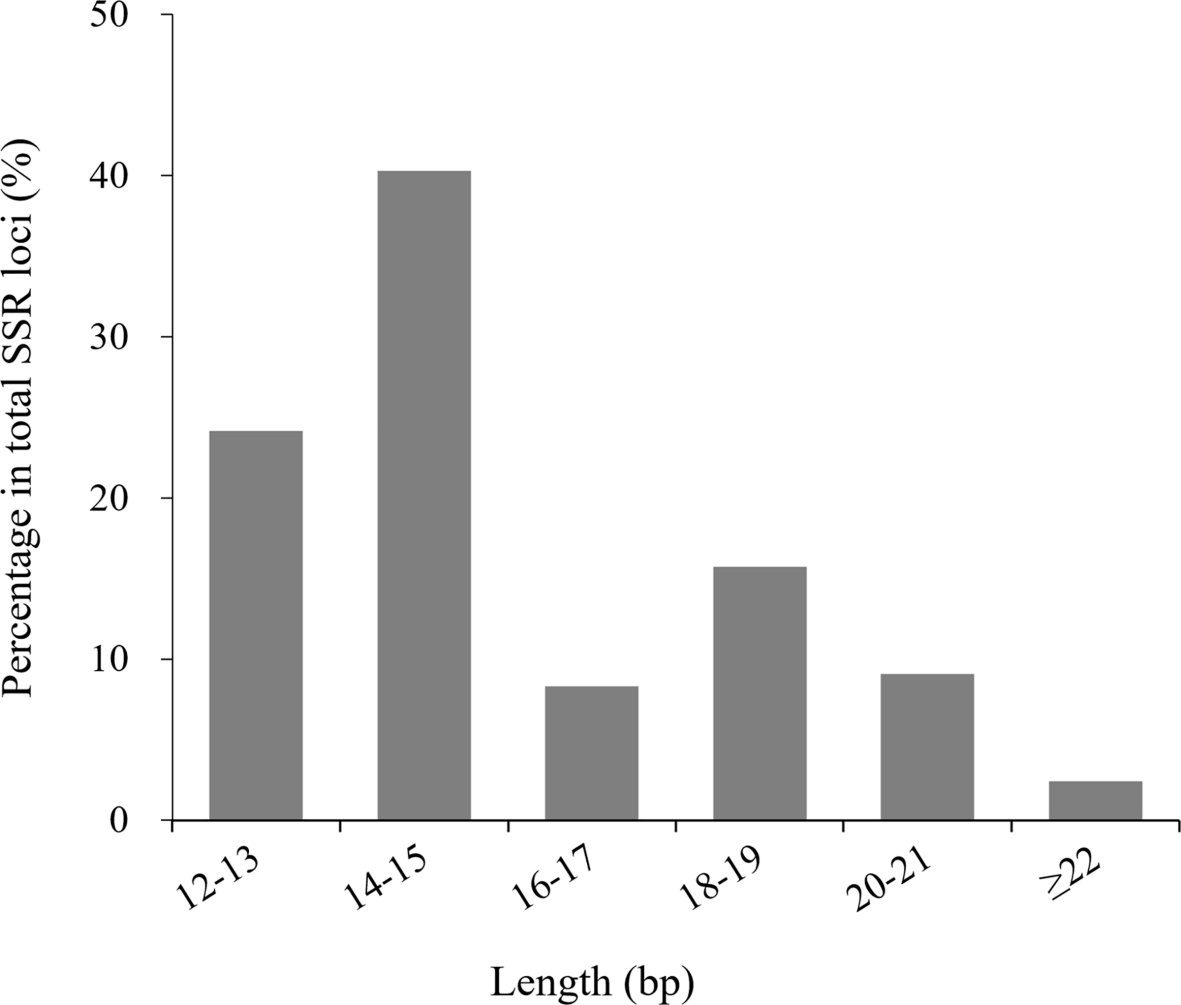
Distribution of the repeat sequence lengths of SSR loci among the transcriptome of flowering Chinese cabbage.

The Fig 4 showed the length distribution of 8,165 SSR loci identified from the transcriptome of flowering Chinese cabbage. These SSR loci could be divided into three groups based on the length of repeat motifs. First group, which was the most abundant and comprised 5,265 SSR loci (64.48%), had repeat motif lengths ≤ 15 bp; second group with 1,962 (24.03%) SSR loci had repeat motif lengths between 16 and 19 bp; and third group, which was the least with 938 (11.49%) SSR loci, had repeat motif lengths ≥ 20 bp.

**Fig 5:**
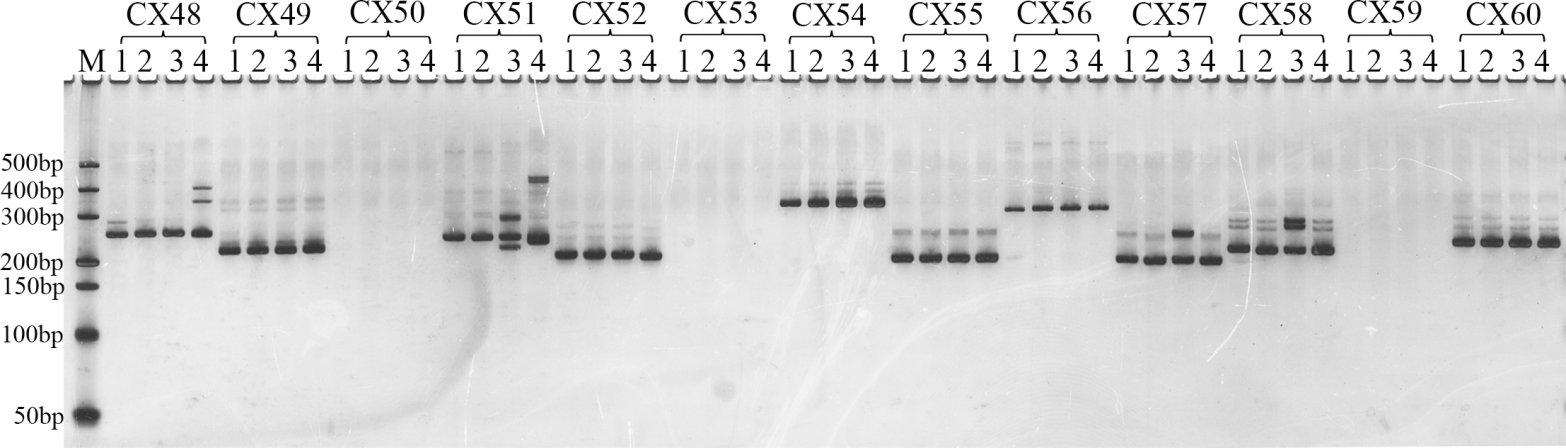
Partial image of the PCR amplification products of 170 selected SSRs in four flowering Chinese cabbage genotypes. Lane M is DNA size marker; lanes 1–4 refer to the four accessions Sijiu-19 caixin, Youlv 50, 3T6 and Liuye 50, respectively; CX48 to CX60 represent SSR primers.

A total of 4,912 primer pairs were designed using BatchPrimer3 software, and 170 (S6 Table) were randomly selected from these loci with repeat motif lengths ≥ 20 bp for PCR using the four flowering Chinese cabbage accessions (Fig 5).

Ninety-six of the 170 tested primer pairs produced clear PCR products, with an amplification efficiency rate of 56.47%. Of these, 41 produced PCR products of the expected fragment size, 22 pairs were shorter and 33 pairs were longer than expected sizes. Forty-eight of the 96 primer pairs that produced PCR showed polymorphism among the four flowering Chinese cabbage accessions (S7 Table). Ten randomly selected PCR products were sequenced, and results showed that all of them contained the corresponding SSR loci.

### Diversity analysis using the validated SSRs

Thirty-four accessions of flowering Chinese cabbage were genotyped using 48 polymorphic SSRs. A total of 213 alleles were scored, and the number of alleles for SSR loci ranged from 2 to 9 with an average of 4.44. The PIC value of these 48 SSRs ranged from 0.19 to 0.85, with an average value of 0.56.

**Fig 6:**
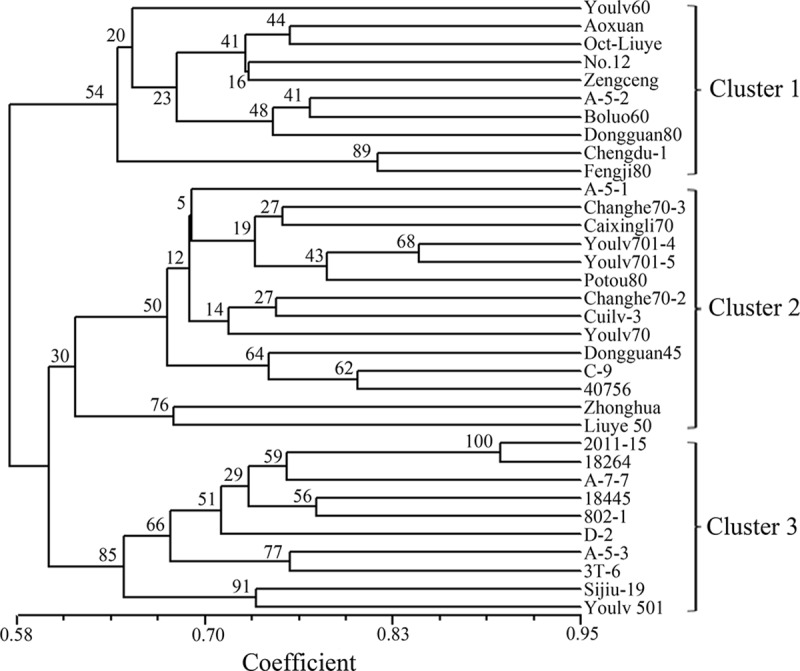
UPGMA dendrogram depicting genetic diversity estimated among 34 flowering Chinese cabbage accessions using 48 SSR markers. The numbers in the dendrogram are bootstrap values.

Genetic similarities of the 34 accessions ranged from 0.46 to 0.90 with an average of 0.62. The largest genetic similarity coefficient (0.90) was observed between “2011–15” and “18264” (Fig 6); whereas the least similarity (0.46) occurred between “No.12” and “802–1”. The 34 accessions could be divided into three clusters with 10, 14 and 10 accessions in cluster 1, 2 and 3, respectively. Cluster 3 was supported by the highest bootstrap value of 85%.

## Discussion

### Transcriptome assembly and functional annotation

*De novo* assembly of short reads of transcriptome without using a reference genome has been widely applied in sequence analysis of non-model organisms recently [24, 25]. In the present study, RNA-seq was performed in flowering Chinese cabbage, a non-model vegetable crop with limited transcriptome data in public databases. We used N50 value and mean length of unigenes as key indicators to evaluate the quality of the assembly [24] and obtained 1,317 bp and 779 bp for these indicators, respectively, in the flowering Chinese cabbage. These values were higher than those in the previous reports for *Lilium Oriental* (N50 = 988 bp and mean length = 673 bp), *Houttuynia cordata* (N50 = 1,051 bp and mean length = 679 bp), and red clover (N50 = 933 bp and mean length = 622 bp) [24, 26, 27], suggesting that our *de novo* transcriptome assembly in flowering Chinese cabbage was effective and accurate.

Of the 48,975 assembled unigenes in flowering Chinese cabbage, 19,599 (40.02%) were longer than 600 bp and 12,733 (26.0%) were longer than 1,000 bp. A total of 37,494 (76.56%) were annotated in Nr database, which was more than those matched for alfalfa (71.8%), *Houttuynia cordata* (62.52%), Tibetan *Sophora moorcroftiana* (47.12%) and mung bean (53.0%) [24, 28, 29, 30]. Unigenes with longer sequences and a higher annotation rate were the indicators of high quality assembly [31, 32] that effectively captured a large portion of the transcriptome.

### SSR loci in the transcriptome of flowering Chinese cabbage

Flowering Chinese cabbage is a special *Brassica* vegetable cultivated mainly in southern China. Unlike other *Brassica* vegetables such as Chinese cabbage, only a few usable SSR markers have been reported in flowering Chinese cabbage. Rapid development of next-generation sequencing technology makes it possible for massive development SSR markers using transcriptome sequencing data [10, 33, 34].

In this study, we generated 48,975 unigenes from 295 million reads by *de novo* assembly. We detected 8,165 SSR loci that represent 16.68% of the generated unigenes. This frequency is higher than a previous report of radish (16.32%) [10], and lower than those in Chinese cabbage (20.16%) and radish (23.79%) [9, 35].

The mean SSR density in flowering Chinese cabbage was 213.9 SSR/Mb, which is higher than the previously reported for radish (202.84 SSR/Mb) [10], and lower than that in Chinese cabbage (255.43 SSR/Mb) [9]. The differences in distribution frequencies and densities of SSR loci may be due to the differences in genomes of different species, the quantity and length of unigenes in the transcriptome data, and the analytic tools used and SSR loci screening conditions [10, 36].

Of the six types of repeat sequence units in the current study, trinucleotide repeats had the highest frequency, followed by dinucleotides and mononucleotides, which agrees with cotton, Chinese cabbage, non-heading Chinese cabbage, and radish [9, 35, 37, 38]. Of the 58 SSR motifs identified in the SSR loci, A/T, AG/CT and AAG/CTT were the richest motifs in mono-, di-, and tri-nucleotide repeat sequence units, respectively, and also the three most abundant motifs in SSR, which agrees with those in Chinese cabbage, non-heading Chinese cabbage and radish [9, 35, 38].

### Validation of EST-SSRs in flowering Chinese cabbage

The levels of polymorphic SSR are reported to vary with the lengths of repeat motifs of SSRs, and the longer the repeat motifs, the higher the possibility of polymorphisms [39, 40]. Therefore we assume that SSRs with repeat motif length ≥ 16 bp are more likely to have polymorphisms. Of 676 designed primers with the repeat motif length ≥ 16 bp, 170 were randomly selected for validation in four flowering Chinese cabbage genotypes. Ninety-six (56.47%) primer pairs produced unambiguous amplicons. The successful amplification rate is higher than those reported in rice bean (21.3%) and cotton (47.7%) [4, 37], but lower than those reported in Chinese cabbage (79.2%) and radish (83.5%) [9, 10]. The possible reasons for low amplification rate might because of at least one primer across a splice site, or amplicons with a large intron or chimeric cDNA contigs [41].

Among the 170 randomly tested primer pairs, 48 (28.24%) were polymorphic in the four flowering Chinese cabbage genotypes, which was slightly lower than those in mung bean (33%) with 31 accessions [30], peanut (40.63%) with six accessions [42], and Chinese cabbage (70.8%) with 24 accessions [9]. However, the polymorphism rate might be affected by numbers of accessions and their genetic backgrounds. For example, Zhang et al. [6] determined the level of polymorphisms for the EST-SSRs developed from sesame transcriptomic data, and found polymorphisms in 32 (11.59%) of the 300 EST-SSRs tested in a panel of 24 cultivated accessions, but the numbers of polymorphic EST-SSRs reached 167 (60.51%) when a wild accession was added to the panel. Thus, the polymorphic level of SSRs in the current study might be higher if the accession number was increased or more diversified germplasm lines were added.

### Genetic diversity in the 34 accessions of flowering Chinese cabbage

All 48 polymorphic SSRs were used to genotype 34 cultivated accessions. A total of 213 allelic variations were detected with an average of 4.44 alleles per SSR. The PIC values of these SSRs ranged from 0.19 to 0.85 with an average of 0.56, which is higher than that reported for mung bean (0.34) [30], but lower than those reported for *Houttuynia cordata* (0.72) and *Hemarthria* (0.71) [24, 43]. A UPGMA dendrogram based on genetic similarity coefficients separated 34 accessions into three distinct clusters, which did not match with their origin or maturity category, but agrees with the previous reports [7, 44] that the genetic basis of the origin and maturity categories were mixed in these cultivated accessions. This may be resulted from a narrow genetic background or limited breeding materials/resources used by breeders as observed in sesame [6]. Therefore, more diverse accessions and alien species should be added to breeding programs to expand the genetic backgrounds of breeding parents of flowering Chinese cabbage. In addition, 6 of the 34 accessions used in the current study (Aoxuan, No.12, Sijiu-19, Youlv 501, Oct-Liuye and Youlv60) were part of a genetic diversity panel used by Guo et al. [7]. They used nine microsatellite-anchored fragment length polymorphism (MFLP) markers to cluster 32 accessions into two groups, with Aoxuan, No.12, Sijiu-19 and Youlv 501 in one group, and Oct-Liuye and Youlv60 in the other. In the current study, Aoxuan, No.12, Oct-Liuye and Youlv60 were clustered in the same group, while Sijiu-19 and Youlv 501 were clustered in another group, indicating that different molecular markers might influence estimates of genetic similarity in flowering Chinese cabbage, which agrees with a previous report [45].

In conclusion, our sequence analysis of transcriptome identified 8,165 EST-SSR loci with 98.57% of 1–3 nucleotide repeats. We designed 4,912 SSR primer pairs and identified 48 polymorphic amplicons in four genotypes from 170 randomly selected primer combinations. Genetic diversity analysis using the 48 markers classified 34 cultivated accessions of flowering Chinese cabbage into three groups. The newly developed EST-SSR markers will be a valuable resource for genetic diversity analysis, QTL mapping and marker-assisted breeding in flowering Chinese cabbage.

## Supporting information

S1 Fig Gene ontology classification of the assembled unigenes. (TIF)

S1 Table Information on the 34 cultivated accessions of flowering Chinese cabbage used for diversity analysis. (DOC)

S2 Table Annotation of all the unigenes. (XLS)

S3 Table KEGG pathways for 6033 unigenes. (DOC)

S4 Table Summary of the SSR investigation. (DOC)

S5 Table Fifty-eight motifs of six types of SSRs and their repeat numbers in the transcriptome of flowering Chinese cabbage. (DOC)

S6 Table 170 SSR primer pairs for testing availability in four flowering Chinese cabbage genotypes. (DOC)

S7 Table Characterization of 48 polymorphic EST-SSRs in the 34 accessions of flowering Chinese cabbage. (DOC)
