# Supplementary figures and images for: Development of EST-SSR markers in flowering Chinese cabbage (Brassica campestris L. ssp. chinensis var. utilis Tsen et Lee) based on de novo transcriptomic assemblies
Source: PLoS One. 2017 Sep 13;12(9):e0184736. doi: 10.1371/journal.pone.0184736 (PMC5597223; doi:10.1371/journal.pone.0184736)

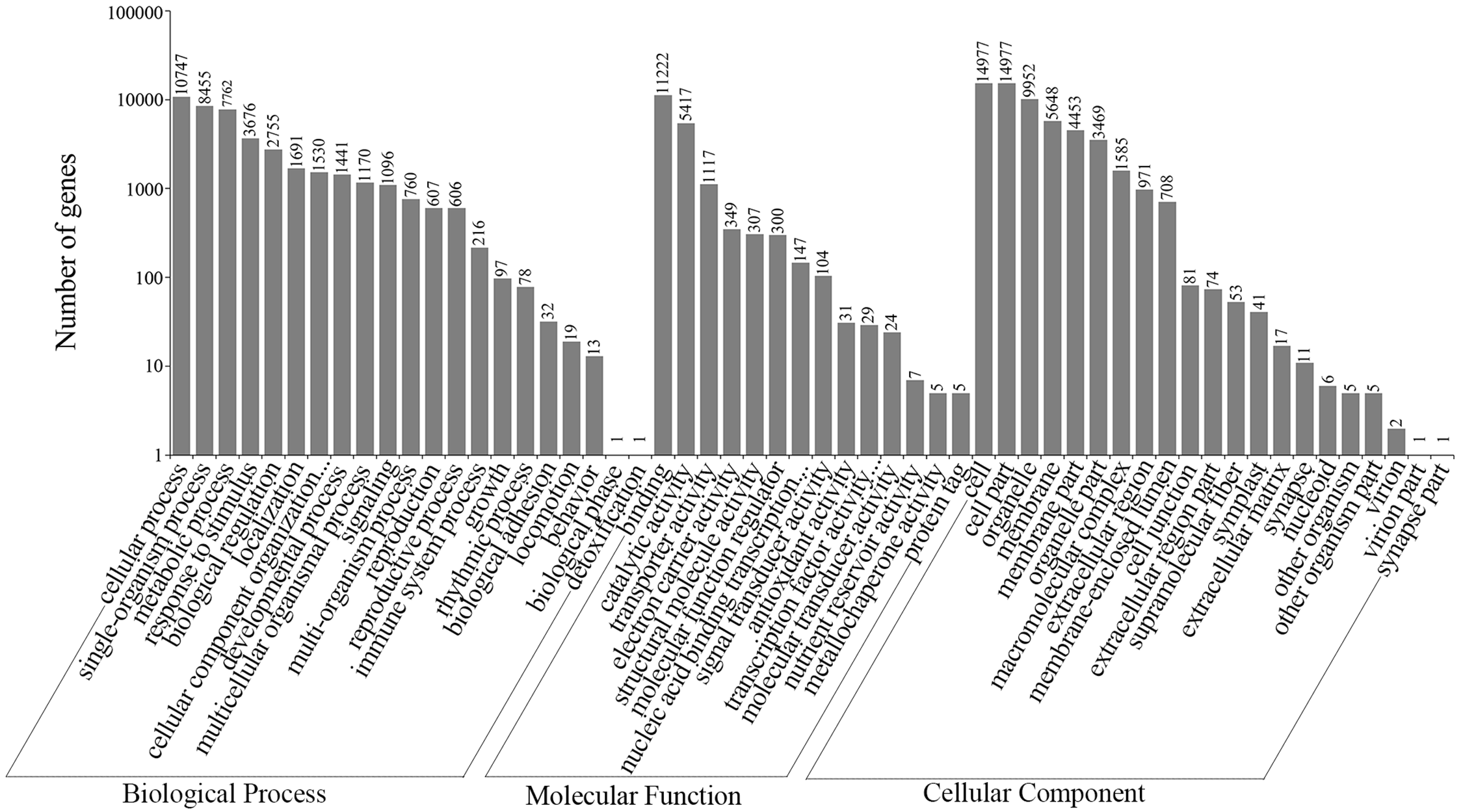

Supplement: S1 Fig — (TIF) [file pone.0184736.s001.tif]
