# Supplementary material for: Development of EST-SSR markers in flowering Chinese cabbage (Brassica campestris L. ssp. chinensis var. utilis Tsen et Lee) based on de novo transcriptomic assemblies
Source: PLoS One. 2017 Sep 13;12(9):e0184736. doi: 10.1371/journal.pone.0184736 (PMC5597223; doi:10.1371/journal.pone.0184736)
Supplement: S1 Table — (DOC) [file pone.0184736.s002.doc]

**S1 Table. Information on the 34 cultivated accessions of flowering Chinese cabbage used for diversity analysis**

| **Maturity category** | **Name** | **Origin** |
| --- | --- | --- |
| Early | Aoxuan | Guangzhou |
|  | No.12 | Guangzhou |
|  | Chengdu-1 | Chengdu |
|  | A-5-2 | Guangzhou |
|  | A-5-1 | Guangzhou |
|  | Cuilv-3 | Guangzhou |
|  | Dongguan45 | Dongguan |
|  | A-5-3 | Guangzhou |
|  | Sijiu-19 | Guangzhou |
|  | Youlv 501 | Guangzhou |
| Mid | Youlv60 | Guangzhou |
|  | Boluo60 | Boluo |
|  | Zhonghua | Guangzhou |
|  | Changhe70-2 | Guangzhou |
|  | Changhe70-3 | Guangzhou |
|  | Caixingli70 | Guangzhou |
|  | Youlv701-4 | Guangzhou |
|  | Youlv701-5 | Guangzhou |
|  | Liuye 50 | Guangzhou |
| Mid–late | Oct-Liuye | Guangxi |
|  | Youlv70 | Guangzhou |
| Late | Fengji80 | Fengji |
|  | Dongguan80 | Dongguan |
|  | 3T-6 | Guangzhou |
|  | Potou80 | Dongguan |
|  | C-9 | Guangzhou |
|  | 42227 | Guangzhou |
|  | 2011-15 | Guangzhou |
|  | A-7-7 | Guangzhou |
|  | 18264 | Guangzhou |
|  | 18445 | Guangzhou |
|  | 802-1 | Guangzhou |
|  | D-2 | Guangzhou |
|  | Zengceng | Zengcheng |
