# Supplementary material for: Development of EST-SSR markers in flowering Chinese cabbage (Brassica campestris L. ssp. chinensis var. utilis Tsen et Lee) based on de novo transcriptomic assemblies
Source: PLoS One. 2017 Sep 13;12(9):e0184736. doi: 10.1371/journal.pone.0184736 (PMC5597223; doi:10.1371/journal.pone.0184736)
Supplement: S3 Table — (DOC) [file pone.0184736.s004.doc]

**S3 Table. KEGG pathways for 6033 unigenes**

| KEGG_A_class | Pathway | Count | Pathway ID |
| --- | --- | --- | --- |
| Organismal Systems | Plant-pathogen interaction | 296 | ko04626 |
|  | Circadian rhythm - plant | 63 | ko04712 |
| Metabolism | Biosynthesis of amino acids | 367 | ko01230 |
|  | Carbon metabolism | 357 | ko01200 |
|  | Starch and sucrose metabolism | 247 | ko00500 |
|  | Purine metabolism | 209 | ko00230 |
|  | Amino sugar and nucleotide sugar metabolism | 184 | ko00520 |
|  | Oxidative phosphorylation | 167 | ko00190 |
|  | Pyrimidine metabolism | 167 | ko00240 |
|  | Phenylpropanoid biosynthesis | 163 | ko00940 |
|  | Cysteine and methionine metabolism | 158 | ko00270 |
|  | Glycolysis / Gluconeogenesis | 133 | ko00010 |
|  | Glutathione metabolism | 119 | ko00480 |
|  | Pyruvate metabolism | 118 | ko00620 |
|  | Glycerophospholipid metabolism | 117 | ko00564 |
|  | Glycine, serine and threonine metabolism | 114 | ko00260 |
|  | 2-Oxocarboxylic acid metabolism | 106 | ko01210 |
|  | Pentose and glucuronate interconversions | 103 | ko00040 |
|  | Glyoxylate and dicarboxylate metabolism | 100 | ko00630 |
|  | Inositol phosphate metabolism | 93 | ko00562 |
|  | Stilbenoid, diarylheptanoid and gingerol biosynthesis | 87 | ko00945 |
|  | Glycerolipid metabolism | 87 | ko00561 |
|  | Fatty acid metabolism | 86 | ko01212 |
|  | Fructose and mannose metabolism | 85 | ko00051 |
|  | Sulfur metabolism | 79 | ko00920 |
|  | Phenylalanine, tyrosine and tryptophan biosynthesis | 78 | ko00400 |
|  | Limonene and pinene degradation | 75 | ko00903 |
|  | Carbon fixation in photosynthetic organisms | 73 | ko00710 |
|  | Arginine and proline metabolism | 71 | ko00330 |
|  | Pentose phosphate pathway | 71 | ko00030 |
|  | Citrate cycle (TCA cycle) | 69 | ko00020 |
|  | Valine, leucine and isoleucine degradation | 68 | ko00280 |
|  | Terpenoid backbone biosynthesis | 67 | ko00900 |
|  | Galactose metabolism | 66 | ko00052 |
|  | Photosynthesis | 64 | ko00195 |
|  | Alanine, aspartate and glutamate metabolism | 63 | ko00250 |
|  | Phenylalanine metabolism | 62 | ko00360 |
|  | Tryptophan metabolism | 61 | ko00380 |
|  | Cyanoamino acid metabolism | 61 | ko00460 |
|  | Porphyrin and chlorophyll metabolism | 60 | ko00860 |
|  | Fatty acid biosynthesis | 56 | ko00061 |
|  | Cutin, suberine and wax biosynthesis | 55 | ko00073 |
|  | N-Glycan biosynthesis | 52 | ko00510 |
|  | Fatty acid degradation | 51 | ko00071 |
|  | Ascorbate and aldarate metabolism | 51 | ko00053 |
|  | Ubiquinone and other terpenoid-quinone biosynthesis | 49 | ko00130 |
|  | Steroid biosynthesis | 45 | ko00100 |
|  | Propanoate metabolism | 45 | ko00640 |
|  | Carotenoid biosynthesis | 44 | ko00906 |
|  | Tyrosine metabolism | 44 | ko00350 |
|  | Arginine biosynthesis | 44 | ko00220 |
|  | alpha-Linolenic acid metabolism | 42 | ko00592 |
|  | Pantothenate and CoA biosynthesis | 42 | ko00770 |
|  | Valine, leucine and isoleucine biosynthesis | 40 | ko00290 |
|  | Glucosinolate biosynthesis | 37 | ko00966 |
|  | Lysine degradation | 37 | ko00310 |
|  | Biosynthesis of unsaturated fatty acids | 36 | ko01040 |
|  | Ether lipid metabolism | 35 | ko00565 |
|  | beta-Alanine metabolism | 34 | ko00410 |
|  | One carbon pool by folate | 33 | ko00670 |
|  | Selenocompound metabolism | 33 | ko00450 |
|  | Fatty acid elongation | 33 | ko00062 |
|  | Nitrogen metabolism | 32 | ko00910 |
|  | Lysine biosynthesis | 32 | ko00300 |
|  | Histidine metabolism | 30 | ko00340 |
|  | Sphingolipid metabolism | 29 | ko00600 |
|  | Flavonoid biosynthesis | 29 | ko00941 |
|  | Nicotinate and nicotinamide metabolism | 27 | ko00760 |
|  | Folate biosynthesis | 26 | ko00790 |
|  | Monobactam biosynthesis | 25 | ko00261 |
|  | Biotin metabolism | 23 | ko00780 |
|  | Diterpenoid biosynthesis | 23 | ko00904 |
|  | Linoleic acid metabolism | 22 | ko00591 |
|  | Vitamin B6 metabolism | 20 | ko00750 |
|  | Tropane, piperidine and pyridine alkaloid biosynthesis | 20 | ko00960 |
|  | Isoquinoline alkaloid biosynthesis | 20 | ko00950 |
|  | Arachidonic acid metabolism | 20 | ko00590 |
|  | Other glycan degradation | 20 | ko00511 |
|  | Thiamine metabolism | 19 | ko00730 |
|  | Butanoate metabolism | 19 | ko00650 |
|  | Zeatin biosynthesis | 19 | ko00908 |
|  | Degradation of aromatic compounds | 18 | ko01220 |
|  | Glycosylphosphatidylinositol(GPI)-anchor biosynthesis | 18 | ko00563 |
|  | Sesquiterpenoid and triterpenoid biosynthesis | 17 | ko00909 |
|  | Indole alkaloid biosynthesis | 16 | ko00901 |
|  | Brassinosteroid biosynthesis | 16 | ko00905 |
|  | Riboflavin metabolism | 13 | ko00740 |
|  | C5-Branched dibasic acid metabolism | 12 | ko00660 |
|  | Photosynthesis - antenna proteins | 12 | ko00196 |
|  | Caffeine metabolism | 11 | ko00232 |
|  | Taurine and hypotaurine metabolism | 11 | ko00430 |
|  | Glycosaminoglycan degradation | 9 | ko00531 |
|  | Glycosphingolipid biosynthesis - ganglio series | 9 | ko00604 |
|  | Monoterpenoid biosynthesis | 7 | ko00902 |
|  | Other types of O-glycan biosynthesis | 6 | ko00514 |
|  | Lipoic acid metabolism | 6 | ko00785 |
|  | Flavone and flavonol biosynthesis | 6 | ko00944 |
|  | Synthesis and degradation of ketone bodies | 5 | ko00072 |
|  | Glycosphingolipid biosynthesis - globo series | 4 | ko00603 |
|  | Betalain biosynthesis | 4 | ko00965 |
|  | Benzoxazinoid biosynthesis | 2 | ko00402 |
|  | Anthocyanin biosynthesis | 1 | ko00942 |
|  | Glycosphingolipid biosynthesis - lacto and neolacto series | 1 | ko00601 |
| Genetic Information Processing | Ribosome | 306 | ko03010 |
|  | Protein processing in endoplasmic reticulum | 278 | ko04141 |
|  | Spliceosome | 251 | ko03040 |
|  | Ubiquitin mediated proteolysis | 240 | ko04120 |
|  | RNA degradation | 186 | ko03018 |
|  | RNA transport | 179 | ko03013 |
|  | mRNA surveillance pathway | 167 | ko03015 |
|  | Ribosome biogenesis in eukaryotes | 133 | ko03008 |
|  | Nucleotide excision repair | 88 | ko03420 |
|  | DNA replication | 87 | ko03030 |
|  | Mismatch repair | 86 | ko03430 |
|  | Homologous recombination | 84 | ko03440 |
|  | Proteasome | 74 | ko03050 |
|  | Aminoacyl-tRNA biosynthesis | 70 | ko00970 |
|  | Protein export | 56 | ko03060 |
|  | SNARE interactions in vesicular transport | 50 | ko04130 |
|  | Base excision repair | 49 | ko03410 |
|  | Basal transcription factors | 45 | ko03022 |
|  | RNA polymerase | 44 | ko03020 |
|  | Sulfur relay system | 21 | ko04122 |
|  | Non-homologous end-joining | 15 | ko03450 |
| Environmental Information Processing | Plant hormone signal transduction | 402 | ko04075 |
|  | Phosphatidylinositol signaling system | 88 | ko04070 |
|  | ABC transporters | 48 | ko02010 |
| Cellular Processes | Endocytosis | 206 | ko04144 |
|  | Peroxisome | 151 | ko04146 |
|  | Phagosome | 123 | ko04145 |
|  | Regulation of autophagy | 32 | ko04140 |
