# Supplementary material for: Development of EST-SSR markers in flowering Chinese cabbage (Brassica campestris L. ssp. chinensis var. utilis Tsen et Lee) based on de novo transcriptomic assemblies
Source: PLoS One. 2017 Sep 13;12(9):e0184736. doi: 10.1371/journal.pone.0184736 (PMC5597223; doi:10.1371/journal.pone.0184736)
Supplement: S4 Table — (DOC) [file pone.0184736.s005.doc]

**S4 Table. Summary of the EST-**SSR investigation

| **Searching items** | **Numbers** |
| --- | --- |
| Total number of examined unigenes | 48,975 |
| Total length of examined sequences (bp) | 38,169,323 |
| Total number of identified EST-SSRs | 8,165 |
| Total number of unigenes containing EST-SSR | 6,778 |
| Number of unigenes containing more than 1 EST-SSR | 1,149 |
| Number of EST-SSRs presented in compound formation | 413 |
