# Supplementary material for: Development of EST-SSR markers in flowering Chinese cabbage (Brassica campestris L. ssp. chinensis var. utilis Tsen et Lee) based on de novo transcriptomic assemblies
Source: PLoS One. 2017 Sep 13;12(9):e0184736. doi: 10.1371/journal.pone.0184736 (PMC5597223; doi:10.1371/journal.pone.0184736)
Supplement: S5 Table — (DOC) [file pone.0184736.s006.doc]

**S5 Table. Fifty-eight motifs of six types of SSRs and their repeat numbers in the transcriptome of flowering Chinese cabbage**

| **Motif** |  |  |  |  | **Repeat number** | | | | | | | | | | | |  |  |  | **Total** |
| --- | --- | --- | --- | --- | --- | --- | --- | --- | --- | --- | --- | --- | --- | --- | --- | --- | --- | --- | --- | --- |
|  | **5** | **6** | **7** | **8** | **9** | **10** | **11** | **12** | **13** | **14** | **15** | **16** | **17** | **18** | **19** | **20** | **21** | **22** | **23** |  |
| A/T | - | - | - | - | - | - | - | 458 | 245 | 153 | 94 | 44 | 39 | 19 | 21 | 27 | 22 | 19 | 12 | 1,153 |
| C/G | - | - | - | - | - | - | - | 5 | 3 | 1 |  | 2 |  |  | 1 | 1 |  |  | 3 | 16 |
| AC/GT | - | 150 | 69 | 57 | 27 | 13 | 2 | 1 | 1 |  |  |  |  |  |  |  |  |  |  | 320 |
| AG/CT | - | 949 | 626 | 473 | 297 | 204 | 68 | 3 |  |  |  |  |  |  |  |  |  |  |  | 2,620 |
| AT/AT | - | 163 | 90 | 63 | 40 | 36 | 21 |  |  |  |  |  |  |  |  |  |  |  |  | 413 |
| CG/CG | - | 1 |  |  |  |  |  |  |  |  |  |  |  |  |  |  |  |  |  | 1 |
| AAC/GTT | 206 | 74 | 49 | 3 | 1 |  |  |  |  |  |  |  |  |  |  |  |  |  |  | 333 |
| AAG/CTT | 778 | 310 | 116 | 3 |  |  |  |  |  |  |  |  |  |  |  |  |  |  |  | 1,207 |
| AAT/ATT | 74 | 21 | 13 | 2 | 1 |  |  |  |  |  |  |  |  |  |  |  |  |  |  | 111 |
| ACC/GGT | 187 | 56 | 31 | 1 |  |  |  |  |  |  |  |  |  |  |  |  |  |  |  | 275 |
| ACG/CGT | 46 | 10 | 2 |  |  |  |  |  |  |  |  |  |  |  |  |  |  |  |  | 58 |
| ACT/AGT | 49 | 16 | 5 |  |  | 1 |  |  |  |  |  |  |  |  |  |  |  |  |  | 71 |
| AGC/CTG | 132 | 61 | 20 | 3 |  |  |  |  |  |  |  |  |  |  |  |  |  |  |  | 216 |
| AGG/CCT | 393 | 150 | 59 | 2 |  |  |  |  |  |  |  |  |  |  |  |  |  |  |  | 604 |
| ATC/ATG | 320 | 154 | 67 | 4 |  |  |  |  |  |  |  |  |  |  |  |  |  |  |  | 545 |
| CCG/CGG | 73 | 27 | 5 |  |  |  |  |  |  |  |  |  |  |  |  |  |  |  |  | 105 |
| AAAC/GTTT | 12 | 1 |  |  |  |  |  |  |  |  |  |  |  |  |  |  |  |  |  | 13 |
| AAAG/CTTT | 22 | 5 |  |  |  |  |  |  |  |  |  |  |  |  |  |  |  |  |  | 27 |
| AAAT/ATTT | 4 | 1 |  |  |  |  |  |  |  |  |  |  |  |  |  |  |  |  |  | 5 |
| AACC/GGTT | 1 | 1 |  |  |  |  |  |  |  |  |  |  |  |  |  |  |  |  |  | 2 |
| AACG/CGTT | 5 | 1 |  |  |  |  |  |  |  |  |  |  |  |  |  |  |  |  |  | 6 |
| AACT/AGTT | 1 |  |  |  |  |  |  |  |  |  |  |  |  |  |  |  |  |  |  | 1 |
| AAGC/CTTG |  |  | 1 |  |  |  |  |  |  |  |  |  |  |  |  |  |  |  |  | 1 |
| AAGG/CCTT | 5 |  |  |  |  |  |  |  |  |  |  |  |  |  |  |  |  |  |  | 5 |
| AAGT/ACTT | 1 |  |  |  |  |  |  |  |  |  |  |  |  |  |  |  |  |  |  | 1 |
| AATC/ATTG | 7 | 1 |  |  |  |  |  |  |  |  |  |  |  |  |  |  |  |  |  | 8 |
| AATT/AATT | 2 |  |  |  |  |  |  |  |  |  |  |  |  |  |  |  |  |  |  | 2 |
| ACAG/CTGT |  | 1 |  |  |  |  |  |  |  |  |  |  |  |  |  |  |  |  |  | 1 |
| ACAT/ATGT | 4 |  |  |  |  |  |  |  |  |  |  |  |  |  |  |  |  |  |  | 4 |
| ACGC/CGTG | 1 |  |  |  |  |  |  |  |  |  |  |  |  |  |  |  |  |  |  | 1 |
| ACGG/CCGT |  |  |  | 1 |  |  |  |  |  |  |  |  |  |  |  |  |  |  |  | 1 |
| ACTC/AGTG | 3 |  |  |  |  |  |  |  |  |  |  |  |  |  |  |  |  |  |  | 3 |
| AGAT/ATCT | 1 | 1 | 1 |  |  |  |  |  |  |  |  |  |  |  |  |  |  |  |  | 3 |
| AGCG/CGCT | 1 |  |  |  |  |  |  |  |  |  |  |  |  |  |  |  |  |  |  | 1 |
| AGGG/CCCT | 2 | 2 |  |  |  |  |  |  |  |  |  |  |  |  |  |  |  |  |  | 4 |
| ATCC/ATGG |  | 2 |  | 1 |  |  |  |  |  |  |  |  |  |  |  |  |  |  |  | 3 |
| AAAAC/GTTTT | 2 |  |  |  |  |  |  |  |  |  |  |  |  |  |  |  |  |  |  | 2 |
| AAAAG/CTTTT | 2 |  |  |  |  |  |  |  |  |  |  |  |  |  |  |  |  |  |  | 2 |
| AAATC/ATTTG | 1 |  |  |  |  |  |  |  |  |  |  |  |  |  |  |  |  |  |  | 1 |
| AACAC/GTGTT |  |  |  | 1 |  |  |  |  |  |  |  |  |  |  |  |  |  |  |  | 1 |
| AACAG/CTGTT | 1 |  |  |  |  |  |  |  |  |  |  |  |  |  |  |  |  |  |  | 1 |
| AACTG/AGTTC | 1 |  |  |  |  |  |  |  |  |  |  |  |  |  |  |  |  |  |  | 1 |
| AAGGG/CCCTT | 1 |  |  |  |  |  |  |  |  |  |  |  |  |  |  |  |  |  |  | 1 |
| AATCG/ATTCG | 2 |  |  |  |  |  |  |  |  |  |  |  |  |  |  |  |  |  |  | 2 |
| AATTC/AATTG | 1 |  |  |  |  |  |  |  |  |  |  |  |  |  |  |  |  |  |  | 1 |
| ACGGG/CCCGT | 1 |  |  |  |  |  |  |  |  |  |  |  |  |  |  |  |  |  |  | 1 |
| AGATG/ATCTC |  | 1 |  |  |  |  |  |  |  |  |  |  |  |  |  |  |  |  |  | 1 |
| ATCCG/ATCGG | 1 |  |  |  |  |  |  |  |  |  |  |  |  |  |  |  |  |  |  | 1 |
| AACCAT/ATGGTT |  | 1 |  |  |  |  |  |  |  |  |  |  |  |  |  |  |  |  |  | 1 |
| AACCGC/CGGTTG | 1 |  |  |  |  |  |  |  |  |  |  |  |  |  |  |  |  |  |  | 1 |
| AACGAG/CGTTCT | 1 |  |  |  |  |  |  |  |  |  |  |  |  |  |  |  |  |  |  | 1 |
| AAGAGG/CCTCTT |  |  | 1 |  |  |  |  |  |  |  |  |  |  |  |  |  |  |  |  | 1 |
| AAGCCC/CTTGGG | 1 |  |  |  |  |  |  |  |  |  |  |  |  |  |  |  |  |  |  | 1 |
| AAGGCC/CCTTGG |  |  |  | 1 |  |  |  |  |  |  |  |  |  |  |  |  |  |  |  | 1 |
| AATGGG/ATTCCC |  | 1 |  |  |  |  |  |  |  |  |  |  |  |  |  |  |  |  |  | 1 |
| ACCATC/ATGGTG | 1 |  |  |  |  |  |  |  |  |  |  |  |  |  |  |  |  |  |  | 1 |
| ACTATC/AGTGAT |  |  |  |  |  | 1 |  |  |  |  |  |  |  |  |  |  |  |  |  | 1 |
| AGGATC/ATCCTG | 1 |  |  |  |  |  |  |  |  |  |  |  |  |  |  |  |  |  |  | 1 |
