# Supplementary material for: Development of EST-SSR markers in flowering Chinese cabbage (Brassica campestris L. ssp. chinensis var. utilis Tsen et Lee) based on de novo transcriptomic assemblies
Source: PLoS One. 2017 Sep 13;12(9):e0184736. doi: 10.1371/journal.pone.0184736 (PMC5597223; doi:10.1371/journal.pone.0184736)
Supplement: S6 Table — (DOC) [file pone.0184736.s007.doc]

**S6 Table. 170 SSR primer pairs for testing availability in four flowering Chinese cabbage genotypes**

| **Primer** | **Primer (5'-3')** | **Tm (°C)** | **Expected size (bp)** | **Motif** | **Repeats** |
| --- | --- | --- | --- | --- | --- |
| CX1 | F: AAGCTCTATGCGCTGATTGC | 60.66 | 220 | TTAT | 5 |
|  | R: TTTTGTCACGGTGTGTTACGA | 60.06 |  |  |  |
| CX2 | F: GGGGCTTAACACCAATGAAA | 59.8 | 185 | AG | 10 |
|  | R: ATCATAGTGCCCCCACACAT | 60.08 |  |  |  |
| CX3 | F: ACTCGAATTCCGGTGAGTTG | 60.11 | 206 | GA | 10 |
|  | R: CATTGCACCTGCTCATGTTT | 59.72 |  |  |  |
| CX4 | F: CTGGAGGCTCTGAGGATGTC | 59.94 | 232 | AGAT | 5 |
|  | R: ACGCCTCCCTTTACCAAGAG | 60.62 |  |  |  |
| CX5 | F: GCGAATCTCCCTCTGAAGAA | 59.51 | 221 | CT | 10 |
|  | R: AAGATTCATGACACTTCAACAAAAA | 59.13 |  |  |  |
| CX6 | F: CCATCTCAAATTTCACAGTAGGA | 58.25 | 302 | TG | 10 |
|  | R: CTGAGATCCCGCCATAAAAA | 60.03 |  |  |  |
| CX7 | F: TCGTTCTCCGGACTCTCTTC | 59.53 | 207 | AG | 10 |
|  | R: CTCAGGGAATGATTGCAAGG | 60.59 |  |  |  |
| CX8 | F: ATGCATTGGTGGAGGAAGAG | 60.07 | 207 | CTTT | 5 |
|  | R: CATTCCATTCCCAAAGATGAA | 59.75 |  |  |  |
| CX9 | F: ACCTCCCCGCTCTCAATAAT | 59.92 | 204 | GA | 10 |
|  | R: TTGGTTCAGCTTTGGGTTTT | 59.59 |  |  |  |
| CX10 | F: ACACGTGGGTTTTCAAGTCC | 59.87 | 229 | AGAA | 5 |
|  | R: CCATTGGATTTGGAGACAGG | 60.31 |  |  |  |
| CX11 | F: TGGGGACCCAAAAGATGTAA | 60.16 | 198 | TTAT | 5 |
|  | R: AACAGAGCAGCAAGCAAACA | 59.79 |  |  |  |
| CX12 | F: AGTTGAAGTCTCCGGCAAGA | 59.99 | 250 | CT | 10 |
|  | R: CCGTGAGAAGTAGACGGATTG | 59.74 |  |  |  |
| CX13 | F: AAAATCAAATCGCCATTTAAAAAC | 59.67 | 186 | CAAA | 5 |
|  | R: AGCTCGGAAAGGTGTTGAGA | 59.99 |  |  |  |
| CX14 | F: AGCTTGTTTGTTTCCGACCA | 60.67 | 193 | GCAC | 5 |
|  | R: GACAGAGAGGGATCAGACGAA | 59.4 |  |  |  |
| CX15 | F: CCAAACCGAAGATCTGGAAA | 60.04 | 199 | AATC | 5 |
|  | R: GAACCACTCCGTGTTCCTGT | 60.01 |  |  |  |
| CX16 | F: CTCTGCTTCAGGGTTTGGAG | 59.98 | 204 | AT | 10 |
|  | R: GCCAAAGTCCTCTTGAGGTT | 58.38 |  |  |  |
| CX17 | F: TTGTTCAAAAATCAAAGAAAAGC | 57.69 | 235 | AT | 10 |
|  | R: CGGCGCTACATATTGGAAGT | 60.12 |  |  |  |
| CX18 | F: GACGATGAGGACGATGACAA | 59.64 | 199 | TTTA | 5 |
|  | R: TTACCGCTCTCAGCTCCTTG | 60.67 |  |  |  |
| CX19 | F: CTTCTCAGCCTCGGTAATGC | 59.98 | 222 | AATC | 5 |
|  | R: CTCCCGACTCACTTCAGGAC | 59.83 |  |  |  |
| CX20 | F: CAGTCGGAGAAGCAATCACA | 59.98 | 258 | AG | 10 |
|  | R: CCACGACTCATGGAAAGTCA | 59.68 |  |  |  |
| CX21 | F: GGACCAACAACCTCACGTTT | 59.87 | 201 | CCTT | 5 |
|  | R: ACTTGATGGTGAGCGCAGAG | 61.57 |  |  |  |
| CX22 | F: CAATGGGAGGAAGAACAAGA | 57.71 | 209 | TC | 10 |
|  | R: CAGAAGGAGCAGAAGCGACT | 59.89 |  |  |  |
| CX23 | F: TCCTTGTCACGTACACACACC | 59.5 | 222 | GATT | 5 |
|  | R: CAGGAGCCACCTTATCTTCG | 59.83 |  |  |  |
| CX24 | F: TGCTCCTCCCATCATGAAAT | 60.43 | 200 | AG | 10 |
|  | R: GAGCTCCACAACGAAACCAC | 60.7 |  |  |  |
| CX25 | F: TTGAGGAACGGATTCAAGGT | 59.53 | 236 | GA | 10 |
|  | R: TCCCAAAGTTTTGTGAAAGGA | 59.57 |  |  |  |
| CX26 | F: GGTTGACCAACAATACTTTGGAA | 60.14 | 264 | TC | 10 |
|  | R: AAGATGCGAATGGGTCTGTC | 60.08 |  |  |  |
| CX27 | F: AAGCCGAGAGCAACGAGTTA | 60.15 | 197 | AACA | 5 |
|  | R: CAGCTTCGTCGGTCCATATC | 60.62 |  |  |  |
| CX28 | F: CACCGGTTGCTGATGAGTAG | 59.31 | 232 | TGTA | 5 |
|  | R: TCTGCTAACTTGCTTGTGCTG | 59.42 |  |  |  |
| CX29 | F: TGCCTTTGTGTTCAGCTCAC | 60.03 | 206 | CA | 10 |
|  | R: CCCAAACGCTTTTGACACAT | 60.92 |  |  |  |
| CX30 | F: TCTCCCACTTGACTTGCACA | 60.44 | 230 | AG | 10 |
|  | R: TGAGTTTATGCTTATCCCATTTGA | 59.87 |  |  |  |
| CX31 | F: GGGAACGGGTTGAGAAAGAT | 60.31 | 234 | ACAA | 5 |
|  | R: GCATCCAAGACGATGTTCAA | 59.65 |  |  |  |
| CX32 | F: CTCGTGAACGAGGTGAAAGA | 59.01 | 206 | AC | 10 |
|  | R: CTTGAGCGTGCTGTGATGTT | 60.06 |  |  |  |
| CX33 | F: TCCATTCAGATTTGGATCCTTC | 60.26 | 208 | CCCT | 5 |
|  | R: CAAGGGGCGGTTCTTTAAGT | 60.47 |  |  |  |
| CX34 | F: CGAGACTCTTGTTGTGTTTGCT | 59.58 | 209 | GTTC | 5 |
|  | R: TTGGCTACGTAAGGGTGAGC | 60.27 |  |  |  |
| CX35 | F: ACTTGGTGTTGTCGCTCTTG | 58.93 | 189 | GA | 10 |
|  | R: AAGACTACAAACAACAGATCCTTCC | 59.17 |  |  |  |
| CX36 | F: TTCCATCACCGTGCTATTCA | 60.07 | 239 | CT | 10 |
|  | R: GCTCTGAGTCCTCAGGGTAGG | 60.4 |  |  |  |
| CX37 | F: TGAAAAGTGAAAACTCTCACCA | 57.51 | 201 | GA | 10 |
|  | R: ATGGCAATGGTCCTGTCTTT | 59.41 |  |  |  |
| CX38 | F: TGAAAAGTGAAAACTCTCACCA | 57.51 | 201 | GA | 10 |
|  | R: ATGGCAATGGTCCTGTCTTT | 59.41 |  |  |  |
| CX39 | F: CAGCTTCTTCTGAACCAAATCA | 59.49 | 202 | GAAC | 5 |
|  | R: TATCGAATCGGTGGAAGGAG | 60.03 |  |  |  |
| CX40 | F: ACCGGGAAAACTAAGGAACC | 59.33 | 219 | AG | 10 |
|  | R: AGGGGAGATGAAGAGGAAGG | 59.63 |  |  |  |
| CX41 | F: TGAGAGAGAGAGAGAGGAATCATTTA | 59.22 | 236 | TCAC | 5 |
|  | R: TTGATGCCTTCCTTCAAATGT | 59.56 |  |  |  |
| CX42 | F: CGTGGTCCGTAGATTTCATTT | 58.96 | 249 | AC | 10 |
|  | R: CCGAGTCAAGTTCTCTGCAA | 59.16 |  |  |  |
| CX43 | F: TGGGGATGTGAGCTTCTTCT | 59.8 | 214 | TA | 10 |
|  | R: AGGGTTCCTTTGGGGTGATA | 60.55 |  |  |  |
| CX44 | F: TGGGTTTTGGGTCAGACATT | 60.21 | 199 | AG | 10 |
|  | R: GCTCGTCTTCGGAGAGATGT | 59.56 |  |  |  |
| CX45 | F: CCGTTACTCAAACCCTTCTCC | 59.98 | 202 | ACCA | 5 |
|  | R: GGGAGAGACAGAACCGAATG | 59.65 |  |  |  |
| CX46 | F: GGACTTTGCCTGCTTCAGTC | 60 | 214 | TC | 10 |
|  | R: TGCAGTGAGGGTCAGACGTA | 60.46 |  |  |  |
| CX47 | F: TCTACCGTTGCTGCTGTTCA | 60.6 | 193 | TA | 10 |
|  | R: GGGGTATAATATCTGCACTTTGG | 58.83 |  |  |  |
| CX48 | F: CAACACAATACAAGAAACAAACAAA | 58.27 | 246 | CTCA | 5 |
|  | R: CGCGAAAGAGAAGTTCGAGT | 59.76 |  |  |  |
| CX49 | F: CCGATCAAAACGGGTTCATA | 60.69 | 210 | ATCA | 5 |
|  | R: TTGCACAGAATTACAACGTTCC | 60.04 |  |  |  |
| CX50 | F: TCCAAAGGGAAAGAGGAACA | 59.64 | 213 | AGA | 7 |
|  | R: AACGAGCTCACCAGAATTGC | 60.41 |  |  |  |
| CX51 | F: TGGAGTGTTTGTTGTAAGCTCAA | 59.84 | 225 | TAA | 7 |
|  | R: TTCGGGATGAGAGTTCCAAG | 60.19 |  |  |  |
| CX52 | F: GTCTCCGGCGATAAAGCATA | 60.2 | 201 | GAG | 7 |
|  | R: TCCTCCTATAGGCCTCGTCA | 59.79 |  |  |  |
| CX53 | F: CCCACTCCATAACAACCAAAA | 59.71 | 206 | AAC | 7 |
|  | R: GCGAGAGTCGTTGAAAGGAA | 60.52 |  |  |  |
| CX54 | F: ATTCAACGACGGATTTGGAG | 59.93 | 206 | TTC | 7 |
|  | R: ACAAACAAAGCATCGGCTCT | 59.88 |  |  |  |
| CX55 | F: CTCCTTGCCTCCACAGTGTT | 60.3 | 183 | TGC | 7 |
|  | R: TAGTCCCATGGGCACAATCT | 60.34 |  |  |  |
| CX56 | F: TTCTTGTCAATCATCAACCTGA | 58.2 | 214 | TGG | 7 |
|  | R: TATCATTCAAGCCACGCACA | 61.22 |  |  |  |
| CX57 | F: TCTTCCGTGTCCCAAGACTC | 60.24 | 186 | GTT | 7 |
|  | R: CGCTGTTCAGAACGAGAGGT | 60.59 |  |  |  |
| CX58 | F: TGACGTGGAGAACAATGTGG | 60.57 | 201 | TAG | 7 |
|  | R: GCAGCTCTTCCAACCAAAAC | 59.86 |  |  |  |
| CX59 | F: ATTTCTCCAGCGTCTTGCTG | 60.54 | 194 | ATA | 7 |
|  | R: CAGCTCTCATCTTCGGGAGT | 59.56 |  |  |  |
| CX60 | F: GAATGGACCAAGGAACTCTCC | 59.93 | 217 | AGA | 7 |
|  | R: GGAACAACCTCACACGCATA | 59.57 |  |  |  |
| CX61 | F: ACAACATCCACGCTCTCCTT | 59.73 | 203 | GAT | 7 |
|  | R: TACTCCGACGTCACCGAGA | 60.41 |  |  |  |
| CX62 | F: CCCCAACAGGCAGAAAAA | 59.62 | 212 | CAA | 7 |
|  | R: CGACCAGATCTGCTGTCTTCT | 59.61 |  |  |  |
| CX63 | F: CAGAACCAGTCGCCACATAA | 59.72 | 202 | GCT | 7 |
|  | R: CTGCTCTCGAGTATGCCTGA | 59.29 |  |  |  |
| CX64 | F: CCGGGTTCCTGATTGTAAAC | 59.29 | 237 | GGA | 7 |
|  | R: GGAAGGCGATAAGAAAGATGG | 60.05 |  |  |  |
| CX65 | F: CAACGCCGAGAAAGATGAAT | 60.21 | 209 | CCT | 7 |
|  | R: TTTCAAGAACCTTGGCTTCG | 60.36 |  |  |  |
| CX66 | F: GTGGTGACGATGTTGATGGT | 59.24 | 211 | GAT | 7 |
|  | R: TCAGCGGCCAGATTCTTTAT | 59.81 |  |  |  |
| CX67 | F: CCTTCTCAGCCTTCTCTCCA | 59.67 | 202 | TCC | 7 |
|  | R: CTTCGTATGCTTCGCTACCC | 59.87 |  |  |  |
| CX68 | F: GCGTGTGTGGTGGTCTGTC | 61.28 | 222 | AGG | 7 |
|  | R: CCTCCGACTCGTGTATCGAC | 60.68 |  |  |  |
| CX69 | F: CCGAAGGTGAAGATCACTGAG | 59.85 | 250 | AAG | 7 |
|  | R: CATTCGCTTTTGTTGGAACC | 60.48 |  |  |  |
| CX70 | F: AGCTGCCTCATCCTGGAGTA | 59.97 | 237 | ATA | 7 |
|  | R: TTGCTCTTGCAAAGGTCTCA | 59.72 |  |  |  |
| CX71 | F: TCGTGAGGTGGTTAACGATG | 59.57 | 198 | GAT | 7 |
|  | R: GCTTCTCTTTCCTTGCAGTCA | 59.75 |  |  |  |
| CX72 | F: GGCTGACGAACCAGAACAAT | 60.12 | 235 | GCA | 7 |
|  | R: ATCTGCTGCTGGCTAATGCT | 60.15 |  |  |  |
| CX73 | F: GCCATCATGGAGGATTTGAT | 59.72 | 211 | GGT | 7 |
|  | R: TTGAAGAAAGAATGCAACAAGTG | 59.43 |  |  |  |
| CX74 | F: ATTCTTCACCGTTCCATCCA | 60.32 | 207 | CAC | 7 |
|  | R: AGGCTTGTGGAAGCTGACAT | 59.87 |  |  |  |
| CX75 | F: ACCCAAGTTGACTGGAGCTG | 60.3 | 197 | GAG | 7 |
|  | R: CGACCAAACCAAGCCATACT | 59.99 |  |  |  |
| CX76 | F: TGGCTTAACGAGCTGGAGAT | 59.98 | 182 | AGC | 7 |
|  | R: CTGATGACGTGAGCTTCCTG | 59.57 |  |  |  |
| CX77 | F: GGAGGTGTTGCTGATGTTGA | 59.68 | 193 | CTG | 7 |
|  | R: CAACTTCAGGCCATGAGAAA | 58.85 |  |  |  |
| CX78 | F: CAACAGCAATGGCTACCTCA | 59.86 | 188 | AAC | 7 |
|  | R: AGGCTTGTTTGGCCATGTTA | 60.5 |  |  |  |
| CX79 | F: CCGACTTAGCCACCGATGTA | 61.04 | 209 | AAC | 7 |
|  | R: GCAGAACTTGGCGCATATCT | 60.38 |  |  |  |
| CX80 | F: TCCTACAACTTCTTGGGGAAA | 58.68 | 168 | ACA | 7 |
|  | R: CCCTGCAATTGAAAACCAGT | 59.97 |  |  |  |
| CX81 | F: TGCCCTTCTTTCATCTGCTT | 59.96 | 200 | CTC | 7 |
|  | R: TCTGTTCCCTCATTCACCAA | 59.06 |  |  |  |
| CX82 | F: CTCCAAATCATACGGCGAAG | 60.6 | 200 | AAC | 7 |
|  | R: TTTCCTTTGTCTATGGTATTTTGCT | 59.51 |  |  |  |
| CX83 | F: TGCTGCTTTTGTCACAGGAC | 60.03 | 214 | ATC | 7 |
|  | R: CGTCTCTTCGACCCTCTTTG | 59.98 |  |  |  |
| CX84 | F: CCGGTACCGAAGAAAATACG | 59.47 | 182 | ATT | 7 |
|  | R: TTTATCCCCAATCCCATGAA | 59.95 |  |  |  |
| CX85 | F: GTTCATCCACATTCGGGTCT | 59.79 | 197 | GAA | 7 |
|  | R: CATCTGACTGCTGAAGCTGAA | 59.33 |  |  |  |
| CX86 | F: ATACAACCCTCAGCCGATTG | 59.96 | 206 | CCA | 7 |
|  | R: TGTGAAGACTGCATCGGAAG | 59.98 |  |  |  |
| CX87 | F: TTCGATTCTTCCTCGATTCC | 59.2 | 200 | GAC | 7 |
|  | R: GTTGCTGCTTCTCCGATCTC | 60.1 |  |  |  |
| CX88 | F: ATTATCGGCTCCCTCTTTCC | 59.51 | 217 | ATT | 7 |
|  | R: AAGCACGAAAGGAGGTTCTG | 59.47 |  |  |  |
| CX89 | F: CGCTAAGCTTCAGACCAAGC | 60.29 | 201 | GTG | 7 |
|  | R: CCACTTTCGCGGTATCTACG | 60.65 |  |  |  |
| CX90 | F: CAAGGGTCTCCTCTGTTCCA | 60.23 | 209 | AAG | 7 |
|  | R: TACCTTCTCCCACGCATCAT | 60.48 |  |  |  |
| CX91 | F: CCCCTCTGCGTCTTCTAATG | 59.83 | 203 | AGA | 7 |
|  | R: CCCTTCTGAAGAATGCGAAA | 60.32 |  |  |  |
| CX92 | F: GGGGTTGCTTGGACTTTCTT | 60.48 | 203 | ATC | 7 |
|  | R: TGCAAAATTCTTGGCTTACTCA | 59.89 |  |  |  |
| CX93 | F: CTGGAGAGAGGGGGTGACTT | 60.64 | 204 | GAG | 7 |
|  | R: GGCTCGTCCATCTCATCTTT | 59.24 |  |  |  |
| CX94 | F: TGCTGCGTTTGCTTCTCTAA | 59.9 | 211 | TCT | 7 |
|  | R: AATGGGATCCGAATCAACAA | 60.13 |  |  |  |
| CX95 | F: CCCCAAATGCATAAGGAAGT | 58.9 | 204 | ACC | 7 |
|  | R: GGTGGTACAAAGCGTGGTCT | 60.03 |  |  |  |
| CX96 | F: CACAGAAGCCCTTCAAGTCC | 59.84 | 200 | TTC | 7 |
|  | R: TTCGGGAGGTGGAAGAATC | 59.99 |  |  |  |
| CX97 | F: CGGAAACTCCTATTCGTGGA | 60.07 | 255 | GAG | 7 |
|  | R: GCCTTCTCCTCAAACACCTCT | 59.87 |  |  |  |
| CX98 | F: GGCGTCTTGTAGGCTGTGTA | 58.95 | 207 | CAT | 7 |
|  | R: GGCGGTTGAGCCTATCTATG | 59.69 |  |  |  |
| CX99 | F: GAGGCGAGAGTCTGTGGTTC | 59.99 | 210 | CGC | 7 |
|  | R: TCAAATCCAACCACCACAAG | 59.39 |  |  |  |
| CX100 | F: CGGAAGAAAATTAAGTGATTCCT | 57.93 | 250 | GAA | 7 |
|  | R: CCGAGATCGATCCAAGCTCT | 61.8 |  |  |  |
| CX101 | F: CAGGAGAAAGGTGGAAGCAA | 60.37 | 193 | CTA | 7 |
|  | R: TCTGCCTCTTCTCATGATGC | 59.07 |  |  |  |
| CX102 | F: TCATGACAAGGATTGCACAAC | 59.56 | 222 | TCT | 7 |
|  | R: AGTAGCACCTCCAAGCCTCA | 60.01 |  |  |  |
| CX103 | F: ACACTGCCTCCTCCCATTCT | 61.05 | 216 | ATC | 7 |
|  | R: CGGATTACCCTCTCGTTGTC | 59.55 |  |  |  |
| CX104 | F: GGAAGGAGTCGTCCCTCTCT | 59.81 | 222 | ATC | 7 |
|  | R: CACTGGTGGTGGTTCTGTTG | 60.04 |  |  |  |
| CX105 | F: CCCAAACAGATCCTTCTCCTC | 60.06 | 211 | CAT | 7 |
|  | R: GTCTGGCTCAGCTTCGTCTT | 59.75 |  |  |  |
| CX106 | F: TGAATGGTCAGAGCGTATTGA | 59.29 | 265 | TCA | 7 |
|  | R: TGCACTATCGTTCCAAGAACA | 59.33 |  |  |  |
| CX107 | F: CAGAGGAGCTCATCCCCTTA | 59.38 | 202 | CAC | 7 |
|  | R: CGAGGCATCACAGTCATCAA | 60.84 |  |  |  |
| CX108 | F: GCTCCGTTCAAGGAGGTTAC | 58.79 | 243 | TTG | 7 |
|  | R: CGCCAGCTCTCAAGAAACAT | 60.54 |  |  |  |
| CX109 | F: GTCGCTGGTACCCCATAGAG | 59.57 | 196 | ATG | 7 |
|  | R: CCATAGCAAACATTGGTGTTG | 58.95 |  |  |  |
| CX110 | F: GGCAGCTTATGTCCAAGCAT | 60.24 | 211 | ATG | 7 |
|  | R: GCATCAGATCCATGGGAAAC | 60.29 |  |  |  |
| CX111 | F: TGCGTTGTGGTCTGAGAATC | 59.84 | 207 | AGA | 7 |
|  | R: AGCCTCACATCAGCGTCTCT | 60.17 |  |  |  |
| CX112 | F: CCCGGAAGAGTTTCCCTATC | 59.9 | 209 | TCA | 7 |
|  | R: TTCCTTCAAAGGGAGCTTCA | 59.93 |  |  |  |
| CX113 | F: CCTTTTCGTTTCCTTGATGTG | 59.6 | 197 | GCC | 7 |
|  | R: GGTGAGACGATGACGAGGAT | 60.08 |  |  |  |
| CX114 | F: CACCCCACCTATACCCAAAA | 59.54 | 294 | TAT | 7 |
|  | R: CATTACTCGTCGATCCCACA | 59.52 |  |  |  |
| CX115 | F: CCGGTGTTGTTTCTCAGGAC | 60.54 | 216 | CTA | 7 |
|  | R: CACCACCCATCAAGAAATCA | 59.34 |  |  |  |
| CX116 | F: GCAGCAGCGAGAACCTTACT | 59.79 | 210 | GGA | 7 |
|  | R: CGAAGCTCTGAGTCATCGAG | 58.85 |  |  |  |
| CX117 | F: AATGGCTTCAGCAAGACCAA | 60.78 | 225 | ACC | 7 |
|  | R: CCACCGATGGGTTTGTTTTA | 60.59 |  |  |  |
| CX118 | F: GCCTGTTGGTTTCTCTACGG | 59.73 | 231 | TAA | 7 |
|  | R: GGACGGAGTTTGAAAGCAAG | 59.85 |  |  |  |
| CX119 | F: GGTGGTTTCGCTAGAGGATG | 59.69 | 211 | GTG | 7 |
|  | R: CTGCGAATTCAACCGTCTCT | 60.4 |  |  |  |
| CX120 | F: CCATCAGCAACAACAACAGC | 60.31 | 200 | ACC | 7 |
|  | R: GGAGATGCAAACCTTCTTCG | 59.81 |  |  |  |
| CX121 | F: TCTCTCCGGATGAAGCAGAT | 59.91 | 192 | GGA | 7 |
|  | R: ATCATCGTTCAGCCAAAACC | 59.94 |  |  |  |
| CX122 | F: CAATCCTCCACGTTCTCGAC | 60.66 | 196 | GGA | 7 |
|  | R: TATCAGCGATCATCCCTTCC | 60 |  |  |  |
| CX123 | F: GTTCCCACAGCTTGGGTTAG | 59.59 | 220 | CCG | 7 |
|  | R: AGGTTATGGAGGTCGCAGTG | 60.13 |  |  |  |
| CX124 | F: GACTTGGGAAGTGGGTCTGA | 60.09 | 204 | GGA | 7 |
|  | R: TAAGGCCGTTCAGATCTTGG | 60.21 |  |  |  |
| CX125 | F: CCCAAGCTCTCGTCTTCTTG | 60.13 | 205 | GGA | 7 |
|  | R: TCCCTCTGTGGAAGCAAATC | 60.19 |  |  |  |
| CX126 | F: TGTCATCAAAATGCCTCCAA | 60.05 | 200 | CTC | 7 |
|  | R: TGTCCTGGAGAATCAAGCAA | 59.37 |  |  |  |
| CX127 | F: CCGCTACTTCCCCTTCTTCT | 59.84 | 203 | GAA | 7 |
|  | R: ATAACGCCTGTCACCAGACC | 60 |  |  |  |
| CX128 | F: TTGCTCAGCAGCACAAAAAC | 60.18 | 183 | ATC | 7 |
|  | R: TGGAGGATAAGGTTGGTTGG | 59.78 |  |  |  |
| CX129 | F: TGTTGGGATCTGAGTGTGGA | 60.09 | 209 | GCT | 7 |
|  | R: GCGAGAGGTTCTGAAGCAAG | 60.28 |  |  |  |
| CX130 | F: CCTCTTCTGTCGGATTCTGC | 59.95 | 195 | GTG | 7 |
|  | R: AGGAGTTTCAGCTGGGGATT | 60.07 |  |  |  |
| CX131 | F: AGGGAAATTTAGGGCGTTTC | 59.44 | 223 | TGC | 7 |
|  | R: TATTCCAGATTTGCCTGCAC | 58.72 |  |  |  |
| CX132 | F: CCACCGTGGATTTTAGAGGA | 59.93 | 224 | GGT | 7 |
|  | R: CGAAGACTGTCTCGCCAAG | 59.71 |  |  |  |
| CX133 | F: GGAGGAGGATGGAGATGGAT | 60.24 | 195 | TGA | 7 |
|  | R: ACTTGTGGCACACCATAACG | 59.49 |  |  |  |
| CX134 | F: TAAGGCTACCACGGTCGTTT | 59.63 | 242 | AGG | 7 |
|  | R: CCCCTTTTGTCGTCTTACCA | 59.96 |  |  |  |
| CX135 | F: CCGATGTTGGAGGAGTACCA | 60.91 | 207 | TTC | 7 |
|  | R: GCTGCTGTCTTGCTGAGATG | 59.89 |  |  |  |
| CX136 | F: ATCCCACCAAAGATGCTGAG | 60.07 | 197 | TTC | 7 |
|  | R: CGTGGCTGTAGAAGAAGAAGC | 59.28 |  |  |  |
| CX137 | F: TCCTCTTCCCATTCAACGTC | 60.05 | 202 | CAC | 7 |
|  | R: GATACACGGCGTGAGAGGAG | 60.82 |  |  |  |
| CX138 | F: AAGCATACGGAGCATGGATT | 59.56 | 203 | AAG | 7 |
|  | R: TGGGATAGGGATATCGATGG | 59.56 |  |  |  |
| CX139 | F: GCGAAAGCCAAGAAAGAGTT | 58.72 | 189 | AAC | 7 |
|  | R: GAACTCGAACTCGTTGGTCA | 58.85 |  |  |  |
| CX140 | F: CCCGATCTCCTGTTATGCTG | 60.62 | 195 | TGT | 7 |
|  | R: TTCACAACCAAAACCCATCA | 59.79 |  |  |  |
| CX141 | F: GCCCTTTTGACATCCTTCAA | 60.05 | 266 | TGT | 7 |
|  | R: AGATCTAGAGCGGTGGCTGA | 60.12 |  |  |  |
| CX142 | F: GCGAGATGGTTTGAAGGAGA | 60.34 | 244 | GAA | 7 |
|  | R: GGCGGTTAAAGTTCGTGACT | 59.24 |  |  |  |
| CX143 | F: AAGAGATGGCGAGCGTTAGA | 60.12 | 212 | GAA | 7 |
|  | R: TTCTGGAGGGAGAATTTGGA | 59.6 |  |  |  |
| CX144 | F: CGAGCAGCTCTGACTCTCCT | 60.03 | 202 | AAC | 7 |
|  | R: CCACCGTCGTTCTTGTTCTC | 60.69 |  |  |  |
| CX145 | F: CCCCAAATCATTACCAAACG | 60.05 | 251 | CTT | 7 |
|  | R: AAGAAGAGTCGGTGGTGGAG | 59.3 |  |  |  |
| CX146 | F: CCAGAGGATGAGGAAGAGCA | 60.49 | 199 | TGT | 7 |
|  | R: CTCCAGGTCCATCCAACAGT | 59.96 |  |  |  |
| CX147 | F: ATCTCCTGGCATCAATACCG | 59.92 | 234 | TAG | 7 |
|  | R: GTCGCAACGGCTATTCTCAT | 60.24 |  |  |  |
| CX148 | F: GGGCAAGGAGGAAAAGAGAC | 60.19 | 216 | GAT | 7 |
|  | R: CCTGGTAAAACGTGTTGCATT | 59.91 |  |  |  |
| CX149 | F: ACGGTGAAGCTGGAGATGAT | 59.69 | 213 | GAG | 7 |
|  | R: CCCTTCTTCAATGGAGCAAC | 59.67 |  |  |  |
| CX150 | F: CGAAACTCGGCTGAGCTAAC | 60.15 | 206 | GA | 11 |
|  | R: TGTCCTCCCTCTCTCTTCACTT | 59.48 |  |  |  |
| CX151 | F: GAGCAACATTCAGTTGGAGAA | 57.94 | 228 | TA | 11 |
|  | R: TCAGCATTCGATGCTACAAA | 58.02 |  |  |  |
| CX152 | F: AGGATCAAGGGAAGGTTTGC | 60.44 | 209 | GA | 11 |
|  | R: CAAATGTTTTGATCCTTTTCTGG | 59.87 |  |  |  |
| CX153 | F: TGAATGATCCGAAGAAGATCG | 60.16 | 214 | GA | 11 |
|  | R: CATGTCCATGTCTCCATCCA | 60.34 |  |  |  |
| CX154 | F: ACGACGAGACGGAGATGAGT | 59.87 | 199 | AG | 11 |
|  | R: CAAAAGCCATTCTCCCTTTG | 59.68 |  |  |  |
| CX155 | F: TGTTTGCTGATCCAAAAGGA | 59.25 | 193 | AG | 11 |
|  | R: CCAGTTCAAAGCCTTCTTGC | 59.99 |  |  |  |
| CX156 | F: CAACACGCAGCCTAGACAGA | 60.2 | 232 | TCTT | 6 |
|  | R: GGAAGATCCAAGGGAGAGAGA | 59.76 |  |  |  |
| CX157 | F: TCGACGCTGACTTCACTGAC | 60.19 | 200 | GTTG | 6 |
|  | R: GGACAGCTTCACACATTTGC | 59.3 |  |  |  |
| CX158 | F: CCTCTGGTCCTCTGCAATCT | 59.4 | 207 | CAC | 8 |
|  | R: CGACCCACCTTCCTATACCA | 59.81 |  |  |  |
| CX159 | F: ACTCGTCGGGCTCTGAGATA | 59.97 | 202 | TTC | 8 |
|  | R: GATGAGGCTTGTTCCTTCTGA | 59.42 |  |  |  |
| CX160 | F: CTGGGCCTTCTCCATAGCTT | 60.72 | 201 | GGA | 8 |
|  | R: TCCTCCTCCACTCAACCAAC | 60.09 |  |  |  |
| CX161 | F: CTCAAGGTCTGGATGCACAA | 59.83 | 249 | TAA | 8 |
|  | R: GCTGGTGTTATTGTCGGTGA | 59.57 |  |  |  |
| CX162 | F: AACCTCGTTGTCGCTCATGT | 60.72 | 206 | AGC | 8 |
|  | R: TGGGTCAGAAGCTGATTGAA | 59.37 |  |  |  |
| CX163 | F: CTCGAAGAATCGCTGAATGA | 59.1 | 242 | TTTG | 6 |
|  | R: GGCAACAAGCTCCTTTGAGT | 59.48 |  |  |  |
| CX164 | F: TGATCATCACATTAACAGAATTATTGC | 60.47 | 250 | AGAT | 6 |
|  | R: TGCTTTTTCTCCCTCGATTT | 58.9 |  |  |  |
| CX165 | F: AGCTTCATCTGTCGGTCACA | 59.42 | 206 | AAAG | 6 |
|  | R: CCAGTCGGAATCGTCTTCTC | 59.8 |  |  |  |
| CX166 | F: GGGAGAAGGTTCATGGTTTG | 59.38 | 193 | TTG | 8 |
|  | R: TTCAATCAACACACAAAGATTGG | 59.89 |  |  |  |
| CX167 | F: TCAGACCTAACGAGCGATCA | 59.55 | 210 | CTTT | 6 |
|  | R: TGGTGATGACGAAAGCTTCA | 60.39 |  |  |  |
| CX168 | F: GGCATGTACCCCATTGTAGG | 60.07 | 205 | TGT | 8 |
|  | R: GCAGCTGCTCAGAACGCTAT | 60.85 |  |  |  |
| CX169 | F: GTGGGGATGCTGACAGAGTA | 58.67 | 210 | AAT | 9 |
|  | R: TCCCTGAGAACTAAGGCATCA | 59.82 |  |  |  |
| CX170 | F: AACGATCATACAAATTATGTCTCAAA | 58.18 | 205 | TAG | 10 |
|  | R: GGTGCATCACATTCATGTTCA | 60.39 |  |  |  |
