# Supplementary material for: Development of EST-SSR markers in flowering Chinese cabbage (Brassica campestris L. ssp. chinensis var. utilis Tsen et Lee) based on de novo transcriptomic assemblies
Source: PLoS One. 2017 Sep 13;12(9):e0184736. doi: 10.1371/journal.pone.0184736 (PMC5597223; doi:10.1371/journal.pone.0184736)
Supplement: S7 Table — (DOC) [file pone.0184736.s008.doc]

**S7 Table. Characterization of 48 polymorphic EST-SSRs** in the 34 accessions of flowering Chinese cabbage

| **Primer number** | **Primer sequence (5'→3')** | **Expected length/bp** | **Motif** | **Repeats** | **Na** | ***He*** | ***Ho*** | **PIC** |
| --- | --- | --- | --- | --- | --- | --- | --- | --- |
| CX3 | F: ACTCGAATTCCGGTGAGTTG | 206 | GA | 10 | 7 | 0.81 | 0.97 | 0.80 |
|  | R: CATTGCACCTGCTCATGTTT |  |  |  |  |  |  |  |
| CX4 | F: CTGGAGGCTCTGAGGATGTC | 232 | AGAT | 5 | 2 | 0.46 | 0.00 | 0.35 |
|  | R: ACGCCTCCCTTTACCAAGAG |  |  |  |  |  |  |  |
| CX9 | F: ACCTCCCCGCTCTCAATAAT | 204 | GA | 10 | 5 | 0.78 | 0.85 | 0.71 |
|  | R: TTGGTTCAGCTTTGGGTTTT |  |  |  |  |  |  |  |
| CX11 | F: TGGGGACCCAAAAGATGTAA | 198 | TTAT | 5 | 3 | 0.54 | 0.24 | 0.52 |
|  | R: AACAGAGCAGCAAGCAAACA |  |  |  |  |  |  |  |
| CX12 | F: AGTTGAAGTCTCCGGCAAGA | 250 | CT | 10 | 2 | 0.46 | 0.00 | 0.35 |
|  | R: CCGTGAGAAGTAGACGGATTG |  |  |  |  |  |  |  |
| CX13 | F: AAAATCAAATCGCCATTTAAAAAC | 186 | CAAA | 5 | 3 | 0.43 | 0.35 | 0.41 |
|  | R: AGCTCGGAAAGGTGTTGAGA |  |  |  |  |  |  |  |
| CX14 | F: AGCTTGTTTGTTTCCGACCA | 193 | GCAC | 5 | 6 | 0.79 | 0.00 | 0.74 |
|  | R: GACAGAGAGGGATCAGACGAA |  |  |  |  |  |  |  |
| CX16 | F: CTCTGCTTCAGGGTTTGGAG | 204 | AT | 10 | 4 | 0.58 | 0.00 | 0.53 |
|  | R: GCCAAAGTCCTCTTGAGGTT |  |  |  |  |  |  |  |
| CX18 | F: GACGATGAGGACGATGACAA | 199 | TTTA | 5 | 4 | 0.58 | 0.03 | 0.51 |
|  | R: TTACCGCTCTCAGCTCCTTG |  |  |  |  |  |  |  |
| CX23 | F: TCCTTGTCACGTACACACACC | 222 | GATT | 5 | 3 | 0.41 | 0.24 | 0.49 |
|  | R: CAGGAGCCACCTTATCTTCG |  |  |  |  |  |  |  |
| CX24 | F: TGCTCCTCCCATCATGAAAT | 200 | AG | 10 | 9 | 0.86 | 1.00 | 0.85 |
|  | R: GAGCTCCACAACGAAACCAC |  |  |  |  |  |  |  |
| CX26 | F: GGTTGACCAACAATACTTTGGAA | 264 | TC | 10 | 8 | 0.88 | 0.97 | 0.85 |
|  | R: AAGATGCGAATGGGTCTGTC |  |  |  |  |  |  |  |
| CX27 | F: AAGCCGAGAGCAACGAGTTA | 197 | AACA | 5 | 2 | 0.46 | 0.00 | 0.35 |
|  | R: CAGCTTCGTCGGTCCATATC |  |  |  |  |  |  |  |
| CX29 | F: TGCCTTTGTGTTCAGCTCAC | 206 | CA | 10 | 8 | 0.83 | 0.91 | 0.78 |
|  | R: CCCAAACGCTTTTGACACAT |  |  |  |  |  |  |  |
| CX32 | F: CTCGTGAACGAGGTGAAAGA | 206 | AC | 10 | 2 | 0.40 | 0.00 | 0.31 |
|  | R: CTTGAGCGTGCTGTGATGTT |  |  |  |  |  |  |  |
| CX33 | F: TCCATTCAGATTTGGATCCTTC | 208 | CCCT | 5 | 2 | 0.44 | 0.00 | 0.34 |
|  | R: CAAGGGGCGGTTCTTTAAGT |  |  |  |  |  |  |  |
| CX39 | F: CAGCTTCTTCTGAACCAAATCA | 202 | GAAC | 5 | 3 | 0.56 | 0.00 | 0.47 |
|  | R: TATCGAATCGGTGGAAGGAG |  |  |  |  |  |  |  |
| CX41 | F: TGAGAGAGAGAGAGAGGAATCATTTA | 236 | TCAC | 5 | 3 | 0.30 | 0.24 | 0.32 |
|  | R: TTGATGCCTTCCTTCAAATGT |  |  |  |  |  |  |  |
| CX42 | F: CGTGGTCCGTAGATTTCATTT | 249 | AC | 10 | 2 | 0.51 | 0.50 | 0.37 |
|  | R: CCGAGTCAAGTTCTCTGCAA |  |  |  |  |  |  |  |
| CX43 | F: TGGGGATGTGAGCTTCTTCT | 214 | TA | 10 | 7 | 0.83 | 0.97 | 0.79 |
|  | R: AGGGTTCCTTTGGGGTGATA |  |  |  |  |  |  |  |
| CX44 | F: TGGGTTTTGGGTCAGACATT | 199 | AG | 10 | 8 | 0.87 | 0.97 | 0.84 |
|  | R: GCTCGTCTTCGGAGAGATGT |  |  |  |  |  |  |  |
| CX45 | F: CCGTTACTCAAACCCTTCTCC | 202 | ACCA | 5 | 6 | 0.84 | 0.74 | 0.79 |
|  | R: GGGAGAGACAGAACCGAATG |  |  |  |  |  |  |  |
| CX46 | F: GGACTTTGCCTGCTTCAGTC | 214 | TC | 10 | 3 | 0.63 | 0.38 | 0.57 |
|  | R: TGCAGTGAGGGTCAGACGTA |  |  |  |  |  |  |  |
| CX48 | F: CAACACAATACAAGAAACAAACAAA | 246 | CTCA | 5 | 4 | 0.51 | 0.00 | 0.44 |
|  | R: CGCGAAAGAGAAGTTCGAGT |  |  |  |  |  |  |  |
| CX51 | F: TGGAGTGTTTGTTGTAAGCTCAA | 225 | TAA | 7 | 3 | 0.41 | 0.38 | 0.41 |
|  | R: TTCGGGATGAGAGTTCCAAG |  |  |  |  |  |  |  |
| CX57 | F: TCTTCCGTGTCCCAAGACTC | 186 | GTT | 7 | 3 | 0.45 | 0.00 | 0.42 |
|  | R: CGCTGTTCAGAACGAGAGGT |  |  |  |  |  |  |  |
| CX58 | F: TGACGTGGAGAACAATGTGG | 201 | TAG | 7 | 2 | 0.42 | 0.00 | 0.33 |
|  | R: GCAGCTCTTCCAACCAAAAC |  |  |  |  |  |  |  |
| CX62 | F: CCCCAACAGGCAGAAAAA | 212 | CAA | 7 | 8 | 0.82 | 0.97 | 0.81 |
|  | R: CGACCAGATCTGCTGTCTTCT |  |  |  |  |  |  |  |
| CX63 | F: CAGAACCAGTCGCCACATAA | 202 | GCT | 7 | 3 | 0.49 | 0.15 | 0.49 |
|  | R: CTGCTCTCGAGTATGCCTGA |  |  |  |  |  |  |  |
| CX64 | F: CCGGGTTCCTGATTGTAAAC | 237 | GGA | 7 | 3 | 0.63 | 0.00 | 0.55 |
|  | R: GGAAGGCGATAAGAAAGATGG |  |  |  |  |  |  |  |
| CX68 | F: GCGTGTGTGGTGGTCTGTC | 222 | AGG | 7 | 2 | 0.49 | 0.00 | 0.37 |
|  | R: CCTCCGACTCGTGTATCGAC |  |  |  |  |  |  |  |
| CX71 | F: TCGTGAGGTGGTTAACGATG | 198 | GAT | 7 | 3 | 0.52 | 0.00 | 0.45 |
|  | R: GCTTCTCTTTCCTTGCAGTCA |  |  |  |  |  |  |  |
| CX79 | F: CCGACTTAGCCACCGATGTA | 209 | AAC | 7 | 9 | 0.86 | 0.79 | 0.84 |
|  | R: GCAGAACTTGGCGCATATCT |  |  |  |  |  |  |  |
| CX80 | F: TCCTACAACTTCTTGGGGAAA | 168 | ACA | 7 | 3 | 0.55 | 0.00 | 0.45 |
|  | R: CCCTGCAATTGAAAACCAGT |  |  |  |  |  |  |  |
| CX81 | F: TGCCCTTCTTTCATCTGCTT | 200 | CTC | 7 | 9 | 0.87 | 0.62 | 0.85 |
|  | R: TCTGTTCCCTCATTCACCAA |  |  |  |  |  |  |  |
| CX83 | F: TGCTGCTTTTGTCACAGGAC | 214 | ATC | 7 | 2 | 0.21 | 0.00 | 0.19 |
|  | R: CGTCTCTTCGACCCTCTTTG |  |  |  |  |  |  |  |
| CX86 | F: ATACAACCCTCAGCCGATTG | 206 | CCA | 7 | 7 | 0.82 | 0.97 | 0.77 |
|  | R: TGTGAAGACTGCATCGGAAG |  |  |  |  |  |  |  |
| CX93 | F: CTGGAGAGAGGGGGTGACTT | 204 | GAG | 7 | 5 | 0.80 | 0.97 | 0.76 |
|  | R: GGCTCGTCCATCTCATCTTT |  |  |  |  |  |  |  |
| CX111 | F: TGCGTTGTGGTCTGAGAATC | 207 | AGA | 7 | 7 | 0.83 | 0.94 | 0.81 |
|  | R: AGCCTCACATCAGCGTCTCT |  |  |  |  |  |  |  |
| CX112 | F: CCCGGAAGAGTTTCCCTATC | 209 | TCA | 7 | 6 | 0.82 | 0.97 | 0.77 |
|  | R: TTCCTTCAAAGGGAGCTTCA |  |  |  |  |  |  |  |
| CX113 | F: CCTTTTCGTTTCCTTGATGTG | 197 | GCC | 7 | 6 | 0.79 | 0.97 | 0.73 |
|  | R: GGTGAGACGATGACGAGGAT |  |  |  |  |  |  |  |
| CX118 | F: GCCTGTTGGTTTCTCTACGG | 231 | TAA | 7 | 2 | 0.49 | 0.00 | 0.37 |
|  | R: GGACGGAGTTTGAAAGCAAG |  |  |  |  |  |  |  |
| CX119 | F: GGTGGTTTCGCTAGAGGATG | 211 | GTG | 7 | 6 | 0.78 | 0.79 | 0.74 |
|  | R: CTGCGAATTCAACCGTCTCT |  |  |  |  |  |  |  |
| CX128 | F: TTGCTCAGCAGCACAAAAAC | 183 | ATC | 7 | 3 | 0.46 | 0.09 | 0.42 |
|  | R: TGGAGGATAAGGTTGGTTGG |  |  |  |  |  |  |  |
| CX130 | F: CCTCTTCTGTCGGATTCTGC | 195 | GTG | 7 | 2 | 0.40 | 0.00 | 0.31 |
|  | R: AGGAGTTTCAGCTGGGGATT |  |  |  |  |  |  |  |
| CX149 | F: ACGGTGAAGCTGGAGATGAT | 213 | GAG | 7 | 2 | 0.49 | 0.00 | 0.37 |
|  | R: CCCTTCTTCAATGGAGCAAC |  |  |  |  |  |  |  |
| CX157 | F: TCGACGCTGACTTCACTGAC | 200 | GTTG | 6 | 9 | 0.86 | 0.97 | 0.84 |
|  | R: GGACAGCTTCACACATTTGC |  |  |  |  |  |  |  |
| CX162 | F: AACCTCGTTGTCGCTCATGT | 206 | AGC | 8 | 2 | 0.50 | 0.24 | 0.37 |
|  | R: TGGGTCAGAAGCTGATTGAA |  |  |  |  |  |  |  |

**Na =** number of observed alleles; ***He*** **=** number of expected heterozygosity; ***Ho* =** number of observed heterozygosity; **PIC =** polymorphic information content.
